# The crystal structures of six (2*E*)-3-aryl-1-(5-halogeno­thio­phen-2-yl)prop-2-en-1-ones

**DOI:** 10.1107/S2056989015015534

**Published:** 2015-08-26

**Authors:** Vasant S. Naik, Hemmige S. Yathirajan, Jerry P. Jasinski, Victoria A. Smolenski, Christopher Glidewell

**Affiliations:** a Department of Physics, Government First Grade College, Kumta 581 343, India, Research and Development Centre, Bharathiar University, Coimbatore 641 046, India; b Department of Studies in Chemistry, University of Mysore, Manasagangotri, Mysore 570 006, India; c Department of Chemistry, Keene State College, 229 Main Street, Keene, NH 03435-2001, USA; d School of Chemistry, University of St Andrews, St Andrews, Fife KY16 9ST, Scotland

**Keywords:** crystal structure, chalcones, halogenothiophens, hydrogen bonding, halogen⋯halogen inter­actions

## Abstract

The structures of six chalcones containing 5-halogeno­thio­phen-2-yl substituents are reported: (2*E*)-1-(5-chloro­thio­phen-2-yl)-3-(4-ethyl­phen­yl)prop-2-en-1-one, C_15_H_13_ClOS, (I), and (2*E*)-1-(5-bromo­thio­phen-2-yl)-3-(4-ethyl­phen­yl)prop-2-en-1-one, C_15_H_13_BrOS, (II), are isostructural in space group *P*-1, while (2*E*)-1-(5-chloro­thio­phen-2-yl)-3-(4-eth­oxy­phen­yl)prop-2-en-1-one, C_15_H_13_ClO_2_S, (III), and (2*E*)-1-(5-bromo­thio­phen-2-yl)-3-(4-eth­oxy­phen­yl)prop-2-en-1-one C_15_H_13_BrO_2_S, (IV), are isostructural in space group *P*2_1_/*c*. There are no hydrogen bonds of any kind in the structures of compounds (I) and (II), but in the structures of compounds (III) and (IV), the mol­ecules are linked into *C*(7) chains by means of C—H⋯O hydrogen bonds. In the structure of (2*E*)-3-(4-bromo­phen­yl)-1-(5-chloro­thio­phen-2-yl)prop-2-en-1-one, C_13_H_8_BrClOS, (V), there are again no hydrogen bonds nor π–π stacking inter­actions but in that of (2*E*)-1-(5-bromo­thio­phen-2-yl)-3-(3-meth­oxy­phen­yl)prop-2-en-1-one, C_14_H_11_BrO_2_S, (VI), the mol­ecules are linked into *C*(5) chains by C—H⋯O hydrogen bonds. In each of compounds (I)–(VI), the mol­ecular skeletons are close to planarity, and there are short halogen⋯halogen contacts in the structures of compounds (II) and (V) and a short Br⋯O contact in the structure of compound (VI). Comparisons are made with the structures of some similar compounds.

## Abstract 2

Six closely related (2*E*)-3-aryl-1-(5-halogeno­thio­phen-2-yl)prop-2-en-1-ones all have nearly planar mol­ecular skeletons. C—H⋯O hydrogen bonds are present in only three of the structures but short Br⋯Br, Br⋯O and Cl⋯Cl contacts are also present in some of the structures.

## Chemical context   

**Figure 1:**
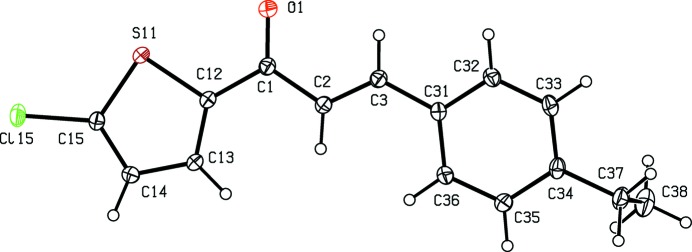
The mol­ecular structure of compound (I), showing the atom-labelling scheme. Displacement ellipsoids are drawn at the 30% probability level.

Chalcones are important constituents of many natural products, and they are abundant in edible plants where they are considered to be precursors of flavonoids and isoflavonoids. They display a wide range of pharmacological properties including anti­bacterial (Tang *et al.*, 2008 ▸; Kumar *et al.*, 2013*a* ▸), anti­cancer (Shin *et al.*, 2013 ▸), anti­fungal (Domínguez *et al.*, 2001 ▸; Kumar *et al.*, 2013*a* ▸,*b* ▸), anti­malarial (Li *et al.*, 1995 ▸) and anti­tubercular (Lin *et al.*, 2002 ▸) activity. In addition, chalcone derivatives are also important materials in photonic applications because of their excellent blue-light transmittance and good crystallization ability (Goto *et al.*, 1991 ▸; Uchida *et al.*,1998 ▸; Indira *et al.*, 2002 ▸; Sarojini *et al.*, 2006 ▸). In a continuation of our work on chalcones containing a thio­phen moiety (Naik *et al.*, 2015 ▸), six new chalcones of this type, compounds (I)–(VI) (Figs. 1 ▸–6 ▸ ▸ ▸ ▸ ▸) have now been synthesized and we report herein on their mol­ecular structures and supra­molecular assembly. Compounds (I)–(VI) were all prepared using condensation reactions, under basic conditions, between 2-acetyl-5-halogeno­thio­phens and substituted benzaldehydes.
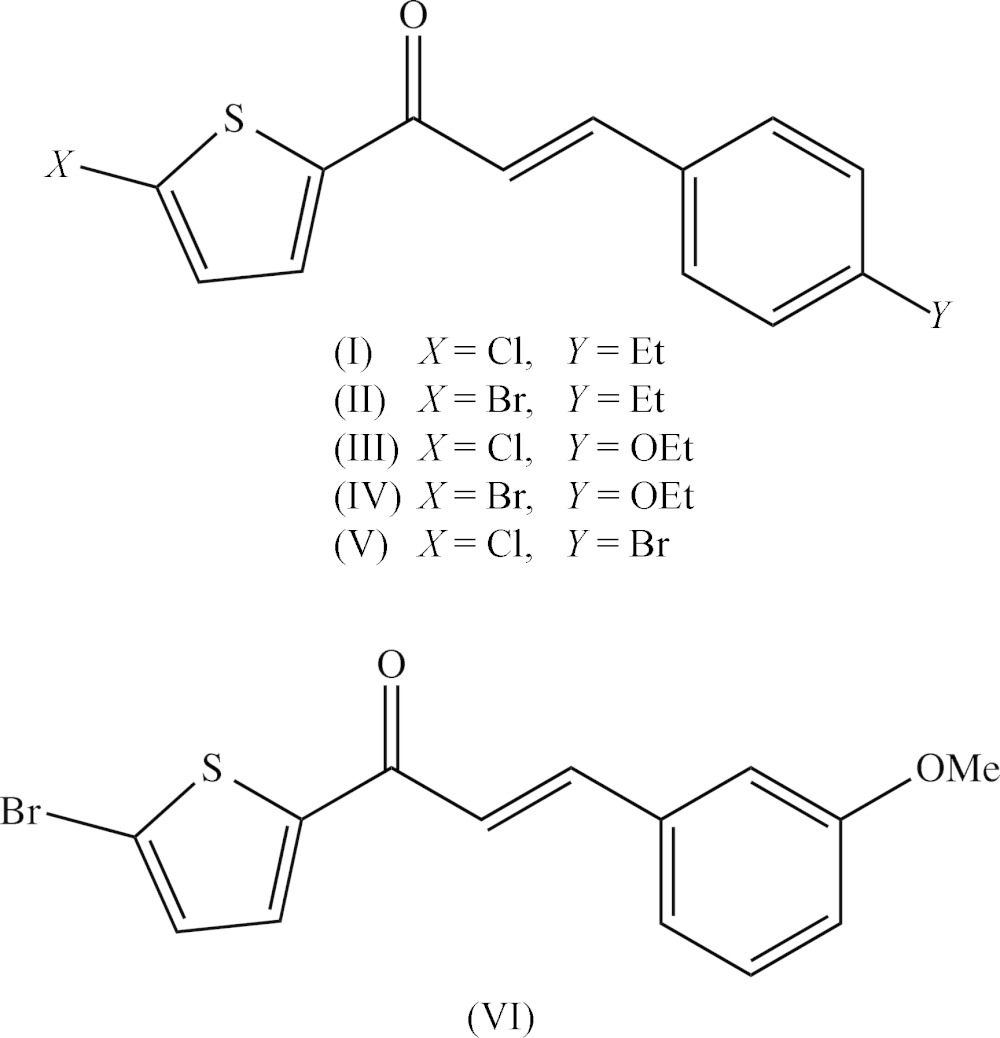


## Structural commentary   

Compounds (I) and (II) are isostructural in space group *P* 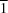, while compounds (III) and (IV) are isostructural in space group *P*2_1_/*c*. Although the unit-cell dimensions for compound (V) are similar to those for compounds (I) and (II), a detailed comparison of the atomic coordinates for compounds (I), (II) and (V) indicates that there is no simple relationship between those of (I) and (II) on the one hand and those of (V) on the other. Although compound (VI) crystallizes in the same space group as compounds (III) and (IV), the unit-cell dimensions for (VI) are very different from those for (III) and (IV).

Although the mol­ecules of compounds (I)–(VI) all lie in general positions, the non-H atoms of the mol­ecular skeletons are quite close to being co-planar, apart from the terminal C atoms of the ethyl groups in compounds (I)–(IV), as shown by the dihedral angles in Table 1 ▸. The values of these angles demonstrate the very close conformational similarity between the mol­ecules of the three compounds, (I), (II) and (V), which crystallize in space group *P* 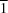, and between those of the three compounds, (III), (IV) and (VI), in *P*2_1_/*c*. In the mol­ecules of each of (I)–(V), the 5-halogenothienyl unit adopts the same orientation, with S11—C12—C1—C2 torsion angles close to 180° (Table 1 ▸). There is no evidence in any of the structures reported here for orientational disorder of the type commonly observed with otherwise unsubstituted thienyl units; this is presumably a direct consequence of the presence of the halogen substituent.

**Figure 3:**
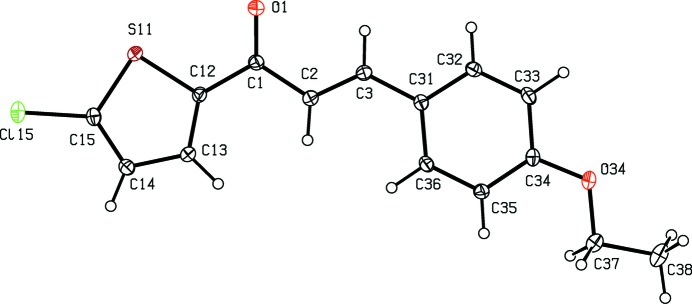
The mol­ecular structure of compound (III), showing the atom-labelling scheme. Displacement ellipsoids are drawn at the 30% probability level.

In each of compounds (III), (IV) and (VI), all of which carry an alk­oxy substituent, the atom C37 (Figs. 3 ▸, 4 ▸ and 6 ▸) lies close to the plane of the adjacent aryl ring: the displacements of the atoms C37 from these planes are 0.117 (3), 0.097 (4) and 0.186 (4) Å, respectively. Consistent with these observations, the corresponding pairs of exocyclic C—C—O angles (Table 1 ▸) differ significantly, as typically found for alk­oxy­benzenes with near-planar mol­ecular skeletons (Seip & Seip, 1973 ▸; Ferguson *et al.*, 1996 ▸). Whereas the whole eth­oxy group in each of compounds (III) and (IV) is nearly coplanar with the adjacent aryl ring, this is far from the case for compounds (I) and (II) (Table 1 ▸, Figs. 1 ▸–4 ▸ ▸ ▸).

The bond distances in compounds (I)–(VI) all lie within the usual ranges (Allen *et al.*, 1987 ▸).

## Supra­molecular inter­actions   

There are no direction-specific inter­molecular inter­actions in the structure of compound (I); hydrogen bonds of C—H⋯O and C—H⋯π types are absent, as are π–π stacking inter­actions. Hydrogen bonds and π–π stacking inter­actions are also absent from the structure of compound (II), but in this structure there is a short inter­molecular Br⋯Br contact, with parameters Br15⋯Br15^i^ = 3.4917 (5) Å and C15—Br15⋯Br15^i^ = 151.37 (8)° [symmetry code: (i) −*x* + 1, −*y* + 1, −*z* + 2]. The Br ⋯Br distance is significantly shorter than the van der Waals contact distance of 3.70 Å (Bondi, 1964 ▸; Rowland & Taylor, 1996 ▸), while the observed C—Br⋯Br angle is consistent with the results of a database analysis of such contacts (Ramasubbu *et al.*, 1986 ▸), which found that such angles were, in general, clustered around 165°.

**Figure 7:**
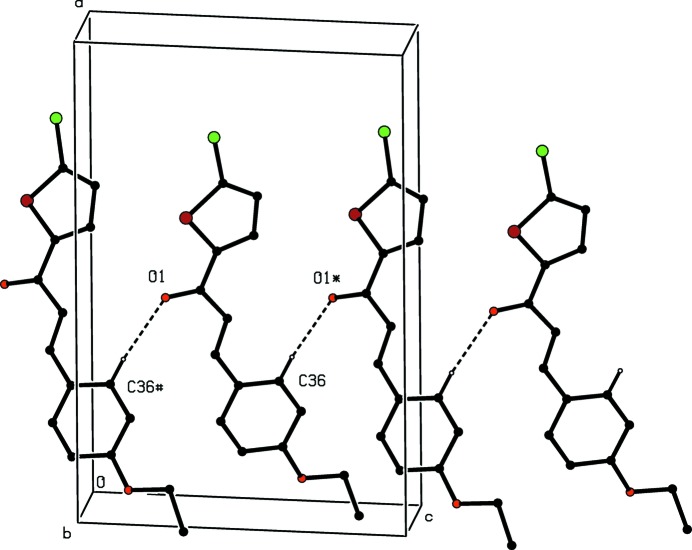
Part of the crystal structure of compound (III), showing the formation of a hydrogen-bonded *C*(7) chain running parallel to the [001] direction. For the sake of clarity, the H atoms not involved in the motif shown have been omitted. The atoms marked with an asterisk (*) or a hash (#) are at the symmetry positions (*x*, −*y* + 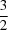, *z* + 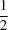) and (*x*, −*y* + 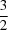, *z* + 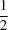), respectively.

In each of compounds (III) and (IV), a single C—H⋯O hydrogen bond having the carbonyl O atom as the acceptor (Table 2 ▸) links mol­ecules related by *c*-glide symmetry into zigzag *C*(7) (Bernstein *et al.*, 1995 ▸) chains running parallel to the [001] direction (Fig. 7 ▸). Two chains of this type, related to one another by inversion, pass through each unit cell, but there are no direction-specific inter­actions between adjacent chains: in particular there are no short inter­molecular Br⋯Br contacts in the structure of compound (IV), thus differing in this respect from compound (II).

There are neither hydrogen bonds nor π–π stacking inter­actions in the structure of compound (V). However, the structure contains a fairly short inter­molecular Cl⋯Cl contact, although, rather surprisingly, there are no short contacts of either Br⋯Br or Br⋯Cl types. For the contact C15—Cl15⋯Cl15^ii^ [symmetry code: (ii) −*x* + 1, −*y*, −*z* + 2], the geometrical parameters are Cl⋯Cl^ii^ = 3.4825 (11) Å and C—Cl⋯Cl^ii^ = 167.83 (10)°. The Cl⋯Cl distances is thus just at the van der Waals contact distance 3.48 Å (Rowland & Taylor, 1996 ▸) and so this contact cannot be regarded as structurally significant: however, it may be noted that the angle C—Cl⋯Cl^ii^ is entirely consistent with the results of a database analysis (Ramasubbu *et al.*, 1986 ▸).

**Figure 8:**
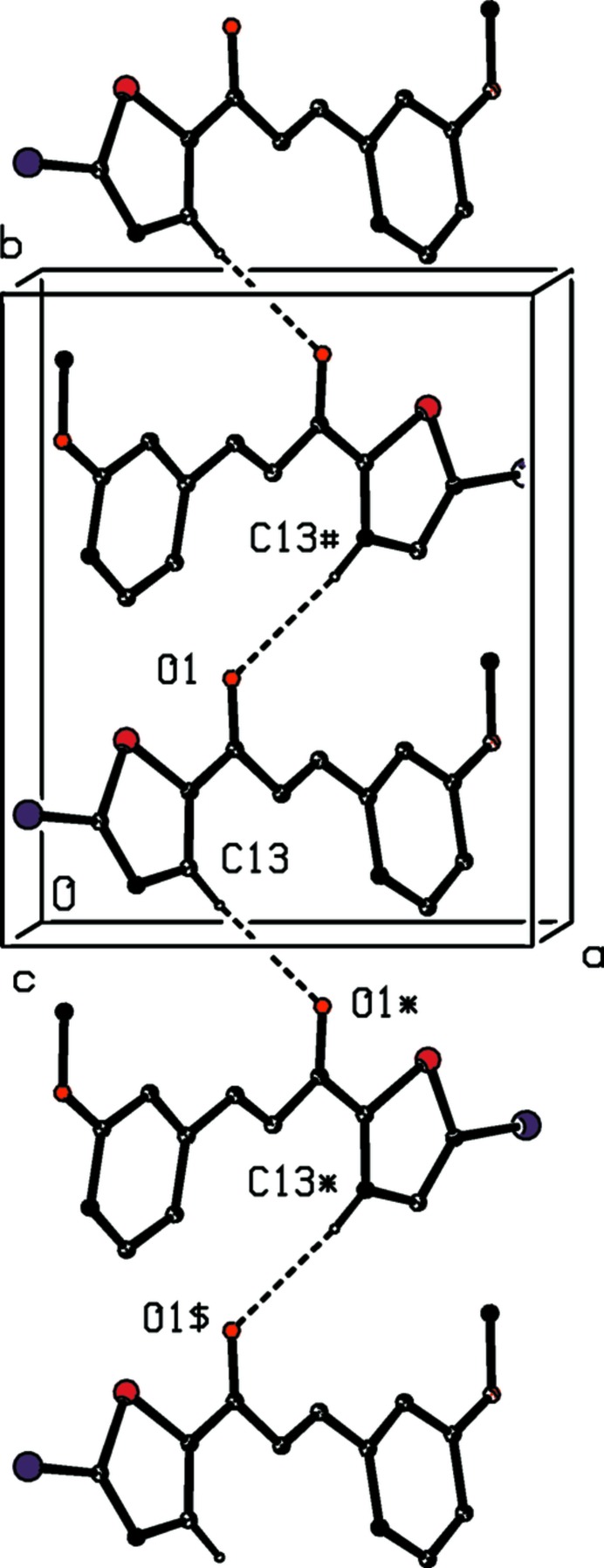
Part of the crystal structure of compound (VI), showing the formation of a hydrogen-bonded *C*(5) chain running parallel to the [010] direction. For the sake of clarity, the H atoms not involved in the motif shown have been omitted. The atoms marked with an asterisk (*), a hash (#) or a dollar sign ($) are at the symmetry positions (−*x* + 1, *y* − 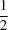, −*z* + 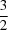), (−*x* + 1, *y* + 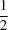, −*z* + 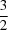) and (*x*, *y* − 1, *z*), respectively.

A single C—H⋯O hydrogen bond (Table 2 ▸) links the mol­ecules of compound (VI) which are related by the 2_1_ screw axis along (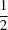, *y*, 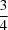) into a *C*(5) chain running parallel to the [010] direction (Fig. 8 ▸). Two chains of this type, related to one another by inversion, pass through each unit cell, but there are no direction-specific inter­actions between adjacent chains. Not only are C—H⋯π hydrogen bonds and π–π stacking inter­actions absent from the crystal structure of compound (VI), but neither are there any short Br⋯Br contacts of the type found in compound (II). There is however a short inter­molecular Br⋯O contact with parameters Br15⋯O33^iii^ = 2.9770 (16) Å and C15—Br15⋯O33^iii^ = 167.21 (7)° [symmetry code: (iii) *x* − , *y*, *z* + 1].

All of the compounds reported here crystallize either in space group *P* 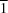 or in *P*2_1_/*c*, and there appear to be some inter­esting connections between the space groups and the nature of the direction-specific inter­molecular inter­actions manifested in the various structures. Thus although all six of the compounds described here contain carbonyl groups, only in compounds (III), (IV) and (VI) do the O atoms of these units participate as acceptors in C—H⋯O hydrogen bonds: these happen to be the three examples which crystallize in space group *P*2_1_/*c*. Of the three 5-bromo­thienyl derivatives reported here, a short Br⋯Br contact occurs only in compound (II), the only example of this group which crystallizes in space group *P* 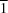.

## Database survey   

The structures of a number of (2*E*)-3-aryl-1-(5-chloro­thio­phen-2-yl)-prop-2-en-1-one derivatives closely related to compounds (I)–(VI) have been reported recently, usually in the form of brief reports on single structures in which no comparisons with related compounds were made, and sometimes with little or no mention of the supra­molecular assembly. It is thus of inter­est briefly to compare the supra­molecular assembly in these compounds with that in compounds (I)–(VI). Compound (VII) (see Scheme below) is isomeric with compound (V), and these two compounds differ only in the exchange of the halogen location. Despite this, they are not isomorphous as compound (VII) crystallizes in space group *P*2_1_/*c* (Kavitha *et al.*, 2013 ▸), as opposed to *P* 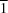 for compound (V). There are two C—H⋯π contacts in the structure of compound (VII), but both of these have long H⋯*D* distances and small *D*—H⋯*A* angles, and so are probably not structurally significant. There is, however, a short inter­molecular Br⋯Cl contact for which the Br⋯Cl distance of 3.5746 (11) Å (not 3.698 (1) Å as stated in the original report), is larger than the sum, 3.55 Å (Rowland & Taylor (1996 ▸), of the van der Waals radii.

**Figure 9:**
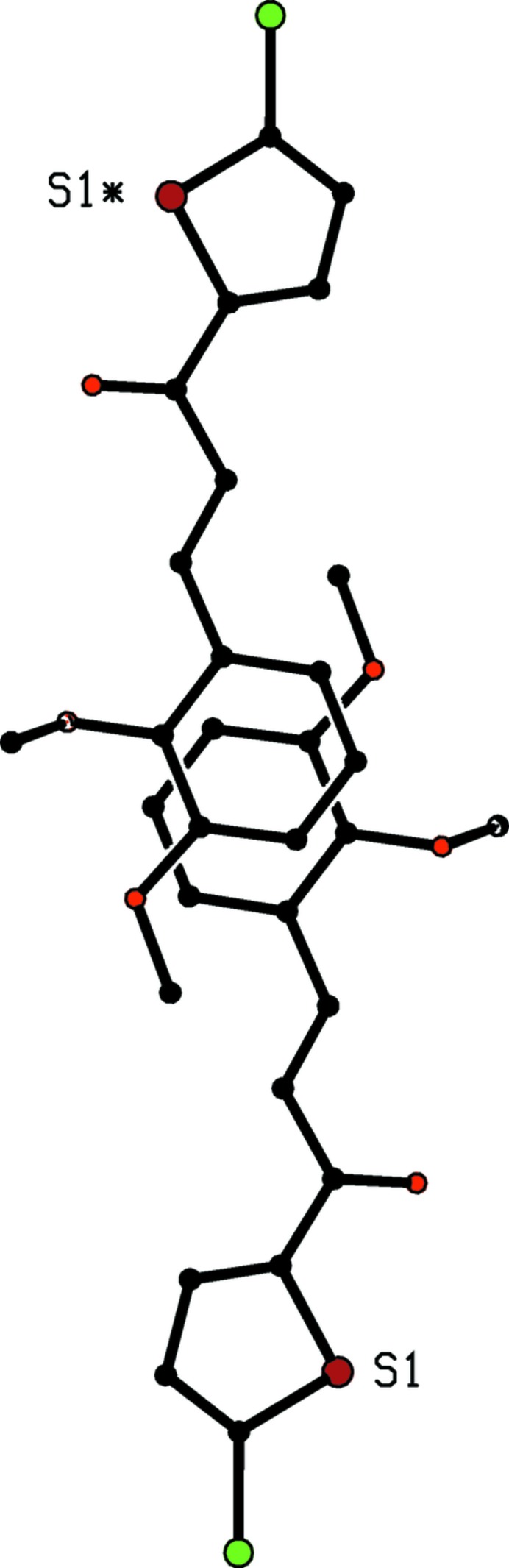
Part of the crystal structure of compound (IX), showing the formation of a centrosymmetric π-stacked dimer. For the sake of clarity, the H atoms and the unit-cell outline have been omitted. The original atomic coordinates (Prabhu *et al.*, 2011*b* ▸) have been used and the S atom marked with an asterisk (*) is at the symmetry position (−*x* + 2, −*y* + 2, −*z* + 2).

For compound (VIII) (Vepuri *et al.*, 2012 ▸), which provides a genuine example of *Z*′ = 2 in space group *Cc* (Baur & Kassner, 1992 ▸; Marsh, 1997 ▸, 2004 ▸), there are no significant direction inter­actions in the structure: in particular there are neither C—H⋯O hydrogen bonds nor short Br⋯Br contacts. Compounds (IX) (Prabhu *et al.*, 2011*b* ▸) and (X) (Prabhu *et al.*, 2014 ▸) are isostructural, and (X) was described as forming chains built from two independent C—H⋯O hydrogen bonds. However, one of these contacts involves a methyl C—H bond and the other has a C—H⋯O angle of only 130° (*cf.* Wood *et al.*, 2009 ▸), so that neither can be regarded as structurally significant. On the other hand the structure of (IX) contains a significant aromatic π–π stacking inter­action between the phenyl rings of inversion-related mol­ecules, although this was apparently overlooked in the original report. The phenyl rings of the mol­ecules at (*x*, *y*, *z*) and (−*x* + 2, −*y* + 2, −z + 2) are strictly parallel with an inter­planar spacing of 3.5113 (8) Å: the ring centroid separation is 3.6535 (11) Å, corresponding to a ring-centroid offset of 1.009 (2) Å, so leading to the formation of a centrosymmetric π-stacked dimer (Fig. 9 ▸).

**Figure 10:**
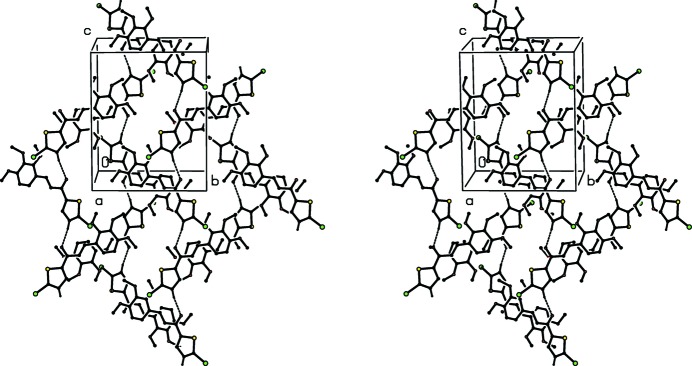
A stereoview of part of the crystal structure of compound (XI), showing the formation of sheets parallel to (100) built from π-stacked hydrogen-bonded *C*(6) chains. The original atomic coordinates (Sunitha *et al.*, 2012 ▸) have been used and, for the sake of clarity, the H atoms not involved in the motif shown have been omitted.

The original report on compound (XI) (Sunitha *et al.*, 2012 ▸) provides no analysis or description of the supra­molecular assembly. Examination of the original atomic coordinates shows firstly that mol­ecules related by a *c*-glide plane are linked by a nearly linear C—H⋯O hydrogen bond, forming a *C*(6) chain running parallel to the [001] direction, and secondly that inversion-related pairs of mol­ecules are linked by a π–π stacking inter­action involving the phenyl rings of the mol­ecules at (*x*, *y*, *z*) and (−*x* + 1, −*y* + 1, −*z*), with inter­planar spacing 3.4465 (10) Å, ring-centroid separation 3.749 (3) Å and ring-centroid offset 1.475 (3) Å. The combined effect of these two types of inter­action is the formation of a sheet lying parallel to (100); see Fig. 10 ▸.
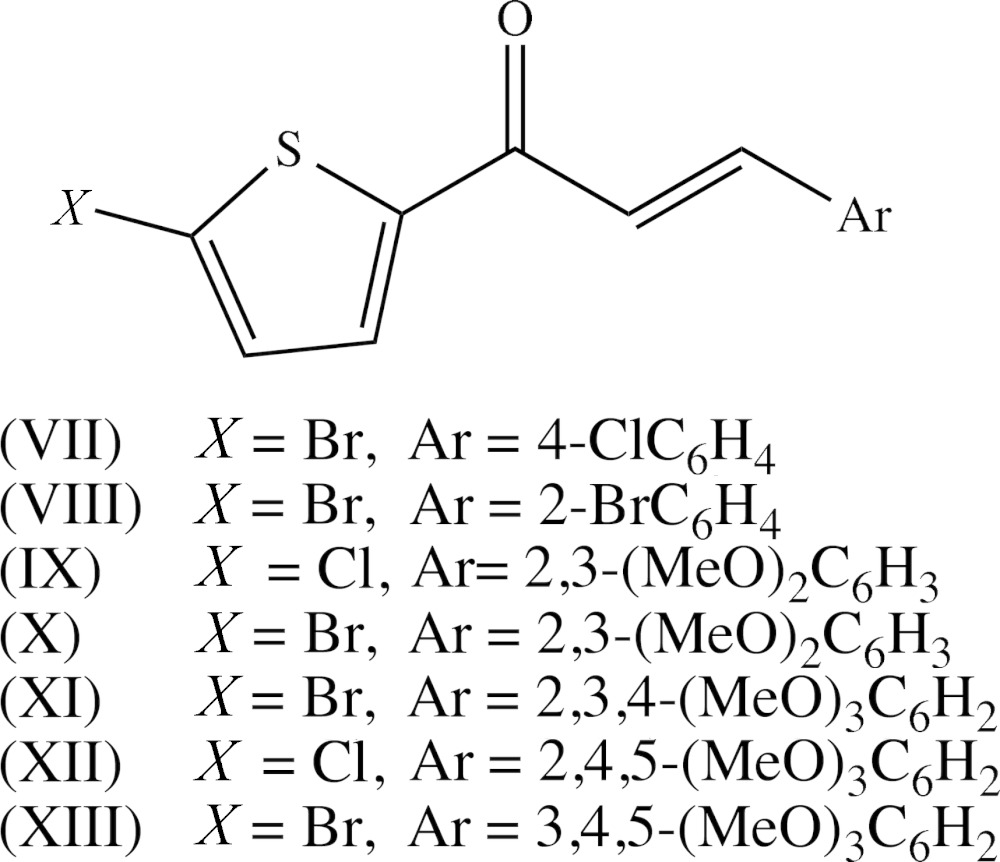


There are two inter­molecular C—H⋯O contacts in the structure of compound (XII) which were described (Prabhu *et al.*, 2011*a* ▸) as joining the mol­ecules into chains: however, for these two contacts the H⋯O distances, 2.68 and 2.71 Å, both exceed the sum of the van der Waals radii, 2.65 Å (Rowland & Taylor, 1996 ▸), so that these contacts certainly cannot be regarded as hydrogen bonds. Simple *C*(11) chains are formed in the structure of compound (XIII) built from C—H⋯O hydrogen bonds (Vepuri *et al.*, 2011 ▸), but there are no short Br⋯Br contacts in either of (XI) and (XIII).

## Synthesis and crystallization   

For the synthesis of each compound, an equimolar mixture (0.01 mol of each component) of the appropriate 2-acetyl-5-halogeno­thio­phen and the appropriately-substituted benz­alde­hyde was dissolved in a mixture of methanol (20 ml) and aqueous sodium hydroxide solution (5 ml of 30% *w*/*v* solution). The mixtures were all stirred at ambient temperature for 4 h, and then poured into ice-cold water (250 ml): the resulting solid products were collected by filtration and dried in air at 323 K. Crystals suitable for single-crystal X-ray diffraction were grown by slow evaporation, at ambient temperature and in the presence of air, of solutions in acetone: melting points: (I) 384 K, (II) 423 K, (III) 415 K. (IV) 403 K, (V) 423 K and (VI) 390 K.

## Refinement   

Crystal data, data collection and structure refinement details are summarized in Table 3 ▸. All H atoms were located in difference Fourier maps and subsequently treated as riding atoms in geometrically idealized positions with C—H distances 0.95 Å (alkenyl, aromatic and heteroaromatic), 0.98 Å (CH_3_) or 0.99 Å (CH_2_), and with *U*_iso_(H) = k*U*_eq_(C), where k = 1.5 for the methyl groups, which were permitted to rotate but not to tilt, and 1.2 for other H atoms. The low-angle reflections (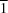,2,1) for compound (III) and (2,1,2) for compound (VI), which had been attenuated by the beam stop, were omitted from the final refinements for these structures.

## Supplementary Material

Crystal structure: contains datablock(s) global, I, II, III, IV, V, VI. DOI: 10.1107/S2056989015015534/su5193sup1.cif

Structure factors: contains datablock(s) I. DOI: 10.1107/S2056989015015534/su5193Isup2.hkl

Structure factors: contains datablock(s) II. DOI: 10.1107/S2056989015015534/su5193IIsup3.hkl

Structure factors: contains datablock(s) III. DOI: 10.1107/S2056989015015534/su5193IIIsup4.hkl

Structure factors: contains datablock(s) IV. DOI: 10.1107/S2056989015015534/su5193IVsup5.hkl

Structure factors: contains datablock(s) V. DOI: 10.1107/S2056989015015534/su5193Vsup6.hkl

Structure factors: contains datablock(s) VI. DOI: 10.1107/S2056989015015534/su5193VIsup7.hkl

Supporting information file. DOI: 10.1107/S2056989015015534/su5193Isup8.cml

Supporting information file. DOI: 10.1107/S2056989015015534/su5193IIsup9.cml

Supporting information file. DOI: 10.1107/S2056989015015534/su5193IIIsup10.cml

Supporting information file. DOI: 10.1107/S2056989015015534/su5193IVsup11.cml

Supporting information file. DOI: 10.1107/S2056989015015534/su5193Vsup12.cml

Supporting information file. DOI: 10.1107/S2056989015015534/su5193VIsup13.cml

CCDC references: 1419530, 1419529, 1419528, 1419527, 1419526, 1419525

Additional supporting information:  crystallographic information; 3D view; checkCIF report

## Figures and Tables

**Figure 2:**
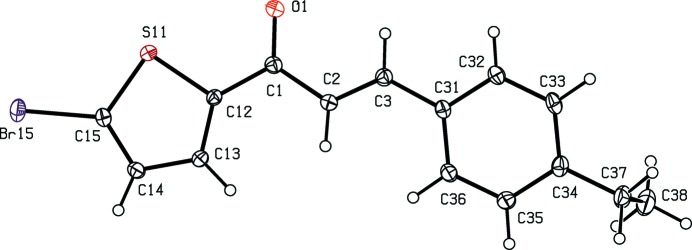
The mol­ecular structure of compound (II), showing the atom-labelling scheme. Displacement ellipsoids are drawn at the 30% probability level.

**Figure 4:**
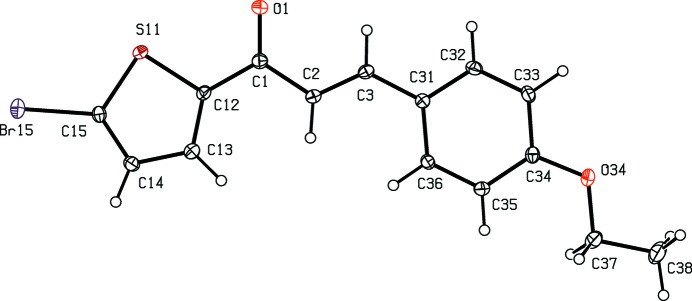
The mol­ecular structure of compound (IV), showing the atom-labelling scheme. Displacement ellipsoids are drawn at the 30% probability level.

**Figure 5:**
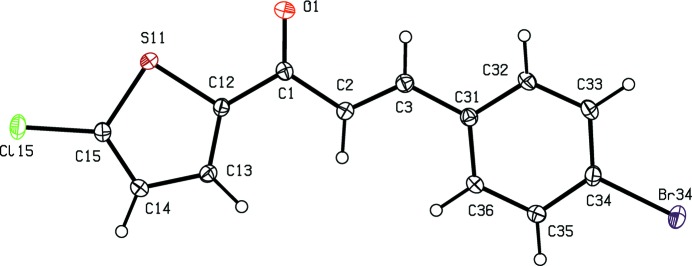
The mol­ecular structure of compound (V), showing the atom-labelling scheme. Displacement ellipsoids are drawn at the 30% probability level.

**Figure 6:**
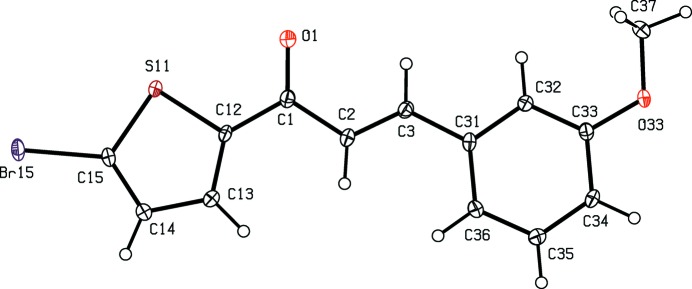
The mol­ecular structure of compound (VI), showing the atom-labelling scheme. Displacement ellipsoids are drawn at the 30% probability level.

**Table 1:** Selected dihedral, bond and torsion angles (°) for compounds (I)–(VI)

Parameter	(I)	(II)	(III)	(IV)	(V)	(VI)
Dihedral 1	2.74 (9)	3.46 (18)	2.80 (14)	3.11 (18)	3.66 (16)	7.70 (13)
Dihedral 2	10.23 (11)	10.5 (2)	3.71 (12)	2.99915)	9.74 (19)	3.10 (11)
Dihedral 3	11.62 (9)	11.78 (16)	6.49 (7)	6.03 (9)	11.96 (14)	5.20 (13)
						
C32—C33—O33						124.89 (19)
C34—C33—O33						114.96 (17)
C33—O33—C37						117.69 (16)
C33—C34—O34			116.33 (13)	116.2 (2)		
C35—C34—O34			123.98 (13)	124.2 (2)		
C34—O34—C37			117.57 (12)	117.45 (18)		
						
S11—C12—C1—C2	178.07 (10)	177.34 (18)	−178.36 (12)	−178.48 (16)	177.99 (19)	−172.61 (16)
C32—C33—O33—C37						7.9 (3)
C33—C34—C37—C38	−97.5 (2)	−92.6 (3)				
C33—C34—O34—C37			−175.99 (15)	−176.45 (19)		
C34—O34—C37—C38			169.60 (16)	171.3 (2)		

‘Dihedral 1’ represents the dihedral angle between the spacer unit (C12,C1,C2,C3,C31) and the thienyl ring. ‘Dihedral 2’ represents the dihedral angle between the spacer unit (C12,C1,C2,C3,C31) and the aryl ring. ‘Dihedral 3’ represents the dihedral angle between the thienyl and aryl rings.

**Table 2:** Hydrogen bond parameters (Å, °) for compounds (III), (IV) and (VI)

Compound	*D*—H⋯*A*	*D*—H	H⋯*A*	*D*⋯*A*	*D*—H⋯*A*
(III)	C36—H36⋯O6^i^	0.95	2.52	3.4649 (18)	173
(IV)	C36—H36⋯O6^i^	0.95	2.52	3.464 (2)	172
(VI)	C13—H13⋯O1^ii^	0.95	2.54	3.446 (3)	159

Symmetry codes: (i) *x*, −*y* + 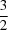, *z* + 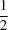; (ii) −*x* + 1, *y* − 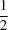, −*z* + 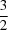.

**Table:** 

	(I)	(II)	(III)
Crystal data			
Chemical formula	C_15_H_13_ClOS	C_15_H_13_BrOS	C_15_H_13_ClO_2_S
*M*_r_	276.76	321.21	292.76
Crystal system, space group	Triclinic, *P* 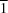	Triclinic, *P* 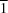	Monoclinic, *P*2_1_/*c*
Temperature (K)	173	173	173
*a*, *b*, *c* (Å)	6.0154 (5), 8.6358 (5), 14.0548 (9)	5.9745 (6), 8.6636 (7), 14.3039 (12)	16.3577 (6), 7.4518 (4), 11.0892 (4)
α, β, γ (°)	74.428 (5), 88.225 (6), 70.417 (6)	74.731 (7), 88.146 (7), 70.334 (8)	90, 92.260 (3), 90
*V* (Å^3^)	661.23 (8)	671.29 (11)	1350.66 (10)
*Z*	2	2	4
Radiation type	Mo *K*α	Mo *K*α	Mo *K*α
μ (mm^−1^)	0.43	3.20	0.43
Crystal size (mm)	0.58 × 0.32 × 0.29	0.45 × 0.22 × 0.16	0.50 × 0.28 × 0.17

Data collection			
Diffractometer	Agilent Eos Gemini	Agilent Eos Gemini	Agilent Eos Gemini
Absorption correction	Multi-scan (*CrysAlis RED*; Agilent, 2012 ▸)	Multi-scan (*CrysAlis RED*; Agilent, 2012 ▸)	Multi-scan (*CrysAlis RED*; Agilent, 2012 ▸)
*T*_min_, *T*_max_	0.750, 0.883	0.326, 0.599	0.789, 0.929
No. of measured, independent and observed [*I* > 2σ(*I*)] reflections	6596, 3861, 3262	6997, 3915, 3089	7925, 3933, 3102
*R*_int_	0.028	0.042	0.038
(sin θ/λ)_max_ (Å^−1^)	0.703	0.703	0.703

Refinement			
*R*[*F* ^2^ > 2σ(*F* ^2^)], *wR*(*F* ^2^), *S*	0.042, 0.119, 1.06	0.041, 0.090, 1.04	0.041, 0.111, 1.06
No. of reflections	3861	3915	3933
No. of parameters	165	165	173
H-atom treatment	H-atom parameters constrained	H-atom parameters constrained	H-atom parameters constrained
Δρ_max_, Δρ_min_ (e Å^−3^)	0.57, −0.34	0.59, −0.63	0.28, −0.39

**Table:** 

	(IV)	(V)	(VI)
Crystal data			
Chemical formula	C_15_H_13_BrO_2_S	C_13_H_8_BrClOS	C_14_H_11_BrO_2_S
*M*_r_	337.21	327.60	323.19
Crystal system, space group	Monoclinic, *P*2_1_/*c*	Triclinic, *P* 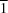	Monoclinic, *P*2_1_/*c*
Temperature (K)	173	173	173
*a*, *b*, *c* (Å)	16.5498 (7), 7.5069 (4), 11.1574 (5)	6.0152 (8), 8.5691 (12), 13.1824 (9)	9.2726 (6), 11.3948 (8), 12.1472 (7)
α, β, γ (°)	90, 92.618 (4), 90	75.25 (1), 81.446 (8), 70.281 (12)	90, 93.273 (6), 90
*V* (Å^3^)	1384.72 (11)	617.09 (14)	1281.37 (14)
*Z*	4	2	4
Radiation type	Mo *K*α	Mo *K*α	Mo *K*α
μ (mm^−1^)	3.11	3.69	3.36
Crystal size (mm)	0.58 × 0.32 × 0.29	0.41 × 0.20 × 0.18	0.54 × 0.42 × 0.31

Data collection			
Diffractometer	Agilent Eos Gemini	Agilent Eos Gemini	Agilent Eos Gemini
Absorption correction	Multi-scan (*CrysAlis RED*; Agilent, 2012 ▸)	Multi-scan (*CrysAlis RED*; Agilent, 2012 ▸)	Multi-scan (*CrysAlis RED*; Agilent, 2012 ▸)
*T*_min_, *T*_max_	0.261, 0.405	0.298, 0.514	0.216, 0.353
No. of measured, independent and observed [*I* > 2σ(*I*)] reflections	8866, 4040, 3189	6674, 3599, 2817	8260, 3722, 2914
*R*_int_	0.037	0.026	0.035
(sin θ/λ)_max_ (Å^−1^)	0.703	0.703	0.703

Refinement			
*R*[*F* ^2^ > 2σ(*F* ^2^)], *wR*(*F* ^2^), *S*	0.038, 0.078, 1.03	0.042, 0.100, 1.03	0.034, 0.075, 1.02
No. of reflections	4040	3599	3722
No. of parameters	174	154	164
H-atom treatment	H-atom parameters constrained	H-atom parameters constrained	H-atom parameters constrained
Δρ_max_, Δρ_min_ (e Å^−3^)	0.54, −0.43	1.43, −0.53	0.49, −0.46

Computer programs: *CrysAlis PRO* and *CrysAlis RED* (Agilent, 2012 ▸), *SHELXS97* (Sheldrick, 2008 ▸), *SHELXL2014* (Sheldrick, 2015 ▸) and *PLATON* (Spek, 2008 ▸).

## supplementary crystallographic information

### (I) (2E)-1-(5-Chlorothiophen-2-yl)-3-(4-ethylphenyl)prop-2-en-1-one Crystal data

**Table:**

C_15_H_13_ClOS	*Z* = 2
*M**_r_* = 276.76	*F*(000) = 288
Triclinic, *P*1	*D*_x_ = 1.390 Mg m^−^^3^
*a* = 6.0154 (5) Å	Mo *K*α radiation, λ = 0.71073 Å
*b* = 8.6358 (5) Å	Cell parameters from 4320 reflections
*c* = 14.0548 (9) Å	θ = 3.4–32.6°
α = 74.428 (5)°	µ = 0.43 mm^−^^1^
β = 88.225 (6)°	*T* = 173 K
γ = 70.417 (6)°	Needle, colourless
*V* = 661.23 (8) Å^3^	0.58 × 0.32 × 0.29 mm

### (I) (2E)-1-(5-Chlorothiophen-2-yl)-3-(4-ethylphenyl)prop-2-en-1-one Data collection

**Table:**

Agilent Eos Gemini diffractometer	3262 reflections with *I* > 2σ(*I*)
Radiation source: Enhance (Mo) X-ray Source	*R*_int_ = 0.028
ω scans	θ_max_ = 30.0°, θ_min_ = 3.4°
Absorption correction: multi-scan (CrysAlis RED; Agilent, 2012)	*h* = −7→8
*T*_min_ = 0.750, *T*_max_ = 0.883	*k* = −11→12
6596 measured reflections	*l* = −19→19
3861 independent reflections	

### (I) (2E)-1-(5-Chlorothiophen-2-yl)-3-(4-ethylphenyl)prop-2-en-1-one Refinement

**Table:**

Refinement on *F*^2^	Hydrogen site location: inferred from neighbouring sites
Least-squares matrix: full	H-atom parameters constrained
*R*[*F*^2^ > 2σ(*F*^2^)] = 0.042	*w* = 1/[σ^2^(*F*_o_^2^) + (0.0573*P*)^2^ + 0.1938*P*] where *P* = (*F*_o_^2^ + 2*F*_c_^2^)/3
*wR*(*F*^2^) = 0.119	(Δ/σ)_max_ = 0.001
*S* = 1.06	Δρ_max_ = 0.57 e Å^−^^3^
3861 reflections	Δρ_min_ = −0.34 e Å^−^^3^
165 parameters	Extinction correction: SHELXL, Fc^*^=kFc[1+0.001xFc^2^λ^3^/sin(2θ)]^-1/4^
0 restraints	Extinction coefficient: 0.019 (5)

### (I) (2E)-1-(5-Chlorothiophen-2-yl)-3-(4-ethylphenyl)prop-2-en-1-one Special details

**Table:**

Geometry. All e.s.d.'s (except the e.s.d. in the dihedral angle between two l.s. planes) are estimated using the full covariance matrix. The cell e.s.d.'s are taken into account individually in the estimation of e.s.d.'s in distances, angles and torsion angles; correlations between e.s.d.'s in cell parameters are only used when they are defined by crystal symmetry. An approximate (isotropic) treatment of cell e.s.d.'s is used for estimating e.s.d.'s involving l.s. planes.

### (I) (2E)-1-(5-Chlorothiophen-2-yl)-3-(4-ethylphenyl)prop-2-en-1-one Fractional atomic coordinates and isotropic or equivalent isotropic displacement parameters (Å^2^)

**Table:**

	*x*	*y*	*z*	*U*_iso_*/*U*_eq_	
C1	0.6919 (3)	0.25997 (18)	0.55004 (11)	0.0231 (3)	
O1	0.9071 (2)	0.23082 (16)	0.54537 (9)	0.0327 (3)	
C2	0.5468 (3)	0.22773 (19)	0.47997 (11)	0.0238 (3)	
H2	0.3798	0.2644	0.4831	0.029*	
C3	0.6465 (3)	0.14771 (18)	0.41211 (11)	0.0240 (3)	
H3	0.8144	0.1055	0.4146	0.029*	
S11	0.73786 (7)	0.36484 (5)	0.71337 (3)	0.02478 (12)	
C12	0.5682 (3)	0.33247 (18)	0.62836 (11)	0.0213 (3)	
C13	0.3333 (3)	0.38074 (19)	0.64782 (11)	0.0240 (3)	
H13	0.2138	0.3719	0.6091	0.029*	
C14	0.2884 (3)	0.4450 (2)	0.73162 (11)	0.0261 (3)	
H14	0.1363	0.4845	0.7557	0.031*	
C15	0.4915 (3)	0.44302 (19)	0.77353 (11)	0.0236 (3)	
Cl15	0.51445 (8)	0.51198 (6)	0.87520 (3)	0.03470 (13)	
C31	0.5211 (3)	0.11904 (18)	0.33424 (11)	0.0224 (3)	
C32	0.6526 (3)	0.03119 (19)	0.26918 (11)	0.0263 (3)	
H32	0.8199	−0.0166	0.2795	0.032*	
C33	0.5411 (3)	0.0132 (2)	0.18973 (12)	0.0282 (3)	
H33	0.6337	−0.0465	0.1464	0.034*	
C34	0.2974 (3)	0.08041 (19)	0.17246 (11)	0.0264 (3)	
C35	0.1656 (3)	0.1642 (2)	0.23891 (12)	0.0292 (3)	
H35	−0.0019	0.2096	0.2291	0.035*	
C36	0.2743 (3)	0.1824 (2)	0.31864 (12)	0.0272 (3)	
H36	0.1806	0.2385	0.3632	0.033*	
C37	0.1759 (4)	0.0704 (2)	0.08306 (13)	0.0344 (4)	
H37A	0.0277	0.0476	0.1022	0.041*	
H37B	0.2794	−0.0259	0.0594	0.041*	
C38	0.1187 (4)	0.2345 (3)	−0.00031 (15)	0.0464 (5)	
H38A	0.0401	0.2232	−0.0568	0.070*	
H38B	0.2653	0.2563	−0.0204	0.070*	
H38C	0.0138	0.3299	0.0225	0.070*	

### (I) (2E)-1-(5-Chlorothiophen-2-yl)-3-(4-ethylphenyl)prop-2-en-1-one Atomic displacement parameters (Å^2^)

**Table:**

	*U*^11^	*U*^22^	*U*^33^	*U*^12^	*U*^13^	*U*^23^
C1	0.0237 (7)	0.0227 (6)	0.0224 (7)	−0.0066 (5)	−0.0010 (5)	−0.0066 (5)
O1	0.0225 (5)	0.0439 (7)	0.0342 (6)	−0.0081 (5)	0.0011 (5)	−0.0188 (5)
C2	0.0226 (7)	0.0257 (7)	0.0240 (7)	−0.0077 (6)	−0.0001 (5)	−0.0085 (6)
C3	0.0240 (7)	0.0246 (7)	0.0234 (7)	−0.0078 (6)	−0.0003 (5)	−0.0067 (5)
S11	0.02067 (19)	0.0317 (2)	0.0247 (2)	−0.00974 (15)	−0.00090 (13)	−0.01084 (15)
C12	0.0220 (7)	0.0211 (6)	0.0211 (6)	−0.0077 (5)	−0.0015 (5)	−0.0057 (5)
C13	0.0218 (7)	0.0289 (7)	0.0248 (7)	−0.0108 (6)	−0.0001 (5)	−0.0102 (6)
C14	0.0223 (7)	0.0310 (7)	0.0279 (8)	−0.0099 (6)	0.0043 (6)	−0.0122 (6)
C15	0.0272 (7)	0.0247 (6)	0.0211 (7)	−0.0104 (6)	0.0009 (5)	−0.0077 (5)
Cl15	0.0419 (2)	0.0431 (2)	0.0267 (2)	−0.01768 (19)	0.00181 (16)	−0.01764 (17)
C31	0.0260 (7)	0.0221 (6)	0.0194 (6)	−0.0084 (6)	0.0016 (5)	−0.0061 (5)
C32	0.0276 (7)	0.0250 (7)	0.0251 (7)	−0.0061 (6)	0.0031 (6)	−0.0086 (6)
C33	0.0358 (8)	0.0268 (7)	0.0261 (7)	−0.0114 (6)	0.0069 (6)	−0.0137 (6)
C34	0.0373 (9)	0.0252 (7)	0.0220 (7)	−0.0161 (6)	0.0021 (6)	−0.0080 (6)
C35	0.0271 (8)	0.0344 (8)	0.0314 (8)	−0.0129 (7)	0.0024 (6)	−0.0144 (7)
C36	0.0268 (7)	0.0322 (7)	0.0269 (7)	−0.0109 (6)	0.0052 (6)	−0.0145 (6)
C37	0.0424 (10)	0.0422 (9)	0.0284 (8)	−0.0219 (8)	0.0006 (7)	−0.0155 (7)
C38	0.0593 (13)	0.0413 (10)	0.0343 (10)	−0.0091 (9)	−0.0109 (9)	−0.0118 (8)

### (I) (2E)-1-(5-Chlorothiophen-2-yl)-3-(4-ethylphenyl)prop-2-en-1-one Geometric parameters (Å, º)

**Table:**

C1—O1	1.2361 (19)	C31—C36	1.401 (2)
C1—C12	1.471 (2)	C32—C33	1.390 (2)
C1—C2	1.474 (2)	C32—H32	0.9500
C2—C3	1.337 (2)	C33—C34	1.386 (2)
C2—H2	0.9500	C33—H33	0.9500
C3—C31	1.466 (2)	C34—C35	1.399 (2)
C3—H3	0.9500	C34—C37	1.509 (2)
S11—C15	1.7105 (16)	C35—C36	1.385 (2)
S11—C12	1.7300 (14)	C35—H35	0.9500
C12—C13	1.374 (2)	C36—H36	0.9500
C13—C14	1.414 (2)	C37—C38	1.519 (3)
C13—H13	0.9500	C37—H37A	0.9900
C14—C15	1.365 (2)	C37—H37B	0.9900
C14—H14	0.9500	C38—H38A	0.9800
C15—Cl15	1.7146 (15)	C38—H38B	0.9800
C31—C32	1.401 (2)	C38—H38C	0.9800
			
O1—C1—C12	119.59 (13)	C33—C32—H32	119.6
O1—C1—C2	123.61 (14)	C31—C32—H32	119.6
C12—C1—C2	116.79 (13)	C34—C33—C32	121.34 (14)
C3—C2—C1	121.02 (14)	C34—C33—H33	119.3
C3—C2—H2	119.5	C32—C33—H33	119.3
C1—C2—H2	119.5	C33—C34—C35	117.93 (14)
C2—C3—C31	126.11 (14)	C33—C34—C37	121.69 (15)
C2—C3—H3	116.9	C35—C34—C37	120.34 (16)
C31—C3—H3	116.9	C36—C35—C34	121.33 (16)
C15—S11—C12	90.59 (7)	C36—C35—H35	119.3
C13—C12—C1	131.06 (13)	C34—C35—H35	119.3
C13—C12—S11	111.68 (11)	C35—C36—C31	120.66 (14)
C1—C12—S11	117.26 (11)	C35—C36—H36	119.7
C12—C13—C14	112.81 (13)	C31—C36—H36	119.7
C12—C13—H13	123.6	C34—C37—C38	111.86 (14)
C14—C13—H13	123.6	C34—C37—H37A	109.2
C15—C14—C13	111.43 (14)	C38—C37—H37A	109.2
C15—C14—H14	124.3	C34—C37—H37B	109.2
C13—C14—H14	124.3	C38—C37—H37B	109.2
C14—C15—S11	113.49 (11)	H37A—C37—H37B	107.9
C14—C15—Cl15	126.35 (13)	C37—C38—H38A	109.5
S11—C15—Cl15	120.16 (9)	C37—C38—H38B	109.5
C32—C31—C36	117.93 (14)	H38A—C38—H38B	109.5
C32—C31—C3	119.01 (14)	C37—C38—H38C	109.5
C36—C31—C3	122.99 (13)	H38A—C38—H38C	109.5
C33—C32—C31	120.75 (15)	H38B—C38—H38C	109.5
			
O1—C1—C2—C3	6.9 (2)	C12—S11—C15—Cl15	−179.17 (9)
C12—C1—C2—C3	−173.77 (13)	C2—C3—C31—C32	−179.46 (15)
C1—C2—C3—C31	−175.39 (13)	C2—C3—C31—C36	3.6 (2)
O1—C1—C12—C13	177.79 (15)	C36—C31—C32—C33	2.2 (2)
C2—C1—C12—C13	−1.6 (2)	C3—C31—C32—C33	−174.90 (13)
O1—C1—C12—S11	−2.53 (19)	C31—C32—C33—C34	−0.3 (2)
C2—C1—C12—S11	178.07 (10)	C32—C33—C34—C35	−1.4 (2)
C15—S11—C12—C13	−0.13 (12)	C32—C33—C34—C37	176.37 (14)
C15—S11—C12—C1	−179.87 (11)	C33—C34—C35—C36	1.1 (2)
C1—C12—C13—C14	179.89 (14)	C37—C34—C35—C36	−176.71 (15)
S11—C12—C13—C14	0.20 (17)	C34—C35—C36—C31	0.9 (2)
C12—C13—C14—C15	−0.18 (19)	C32—C31—C36—C35	−2.5 (2)
C13—C14—C15—S11	0.07 (17)	C3—C31—C36—C35	174.48 (14)
C13—C14—C15—Cl15	179.21 (11)	C33—C34—C37—C38	−97.5 (2)
C12—S11—C15—C14	0.03 (12)	C35—C34—C37—C38	80.2 (2)

### (II) (2E)-1-(5-Bromothiophen-2-yl)-3-(4-ethylphenyl)prop-2-en-1-one Crystal data

**Table:**

C_15_H_13_BrOS	*Z* = 2
*M**_r_* = 321.21	*F*(000) = 324
Triclinic, *P*1	*D*_x_ = 1.589 Mg m^−^^3^
*a* = 5.9745 (6) Å	Mo *K*α radiation, λ = 0.71073 Å
*b* = 8.6636 (7) Å	Cell parameters from 4444 reflections
*c* = 14.3039 (12) Å	θ = 3.4–32.8°
α = 74.731 (7)°	µ = 3.20 mm^−^^1^
β = 88.146 (7)°	*T* = 173 K
γ = 70.334 (8)°	Needle, colourless
*V* = 671.29 (11) Å^3^	0.45 × 0.22 × 0.16 mm

### (II) (2E)-1-(5-Bromothiophen-2-yl)-3-(4-ethylphenyl)prop-2-en-1-one Data collection

**Table:**

Agilent Eos Gemini diffractometer	3089 reflections with *I* > 2σ(*I*)
Radiation source: Enhance (Mo) X-ray Source	*R*_int_ = 0.042
ω scans	θ_max_ = 30.0°, θ_min_ = 3.4°
Absorption correction: multi-scan (CrysAlis RED; Agilent, 2012)	*h* = −6→8
*T*_min_ = 0.326, *T*_max_ = 0.599	*k* = −12→12
6997 measured reflections	*l* = −20→17
3915 independent reflections	

### (II) (2E)-1-(5-Bromothiophen-2-yl)-3-(4-ethylphenyl)prop-2-en-1-one Refinement

**Table:**

Refinement on *F*^2^	Hydrogen site location: inferred from neighbouring sites
Least-squares matrix: full	H-atom parameters constrained
*R*[*F*^2^ > 2σ(*F*^2^)] = 0.041	*w* = 1/[σ^2^(*F*_o_^2^) + (0.0284*P*)^2^] where *P* = (*F*_o_^2^ + 2*F*_c_^2^)/3
*wR*(*F*^2^) = 0.090	(Δ/σ)_max_ < 0.001
*S* = 1.04	Δρ_max_ = 0.59 e Å^−^^3^
3915 reflections	Δρ_min_ = −0.63 e Å^−^^3^
165 parameters	Extinction correction: SHELXL, Fc^*^=kFc[1+0.001xFc^2^λ^3^/sin(2θ)]^-1/4^
0 restraints	Extinction coefficient: 0.0046 (13)

### (II) (2E)-1-(5-Bromothiophen-2-yl)-3-(4-ethylphenyl)prop-2-en-1-one Special details

**Table:**

Geometry. All e.s.d.'s (except the e.s.d. in the dihedral angle between two l.s. planes) are estimated using the full covariance matrix. The cell e.s.d.'s are taken into account individually in the estimation of e.s.d.'s in distances, angles and torsion angles; correlations between e.s.d.'s in cell parameters are only used when they are defined by crystal symmetry. An approximate (isotropic) treatment of cell e.s.d.'s is used for estimating e.s.d.'s involving l.s. planes.

### (II) (2E)-1-(5-Bromothiophen-2-yl)-3-(4-ethylphenyl)prop-2-en-1-one Fractional atomic coordinates and isotropic or equivalent isotropic displacement parameters (Å^2^)

**Table:**

	*x*	*y*	*z*	*U*_iso_*/*U*_eq_	
C1	0.6905 (4)	0.2599 (3)	0.54692 (19)	0.0229 (5)	
O1	0.9055 (3)	0.2306 (3)	0.54185 (14)	0.0324 (4)	
C2	0.5458 (4)	0.2270 (3)	0.47838 (18)	0.0243 (5)	
H2	0.3775	0.2652	0.4813	0.029*	
C3	0.6430 (4)	0.1457 (3)	0.41244 (19)	0.0245 (5)	
H3	0.8121	0.1024	0.4149	0.029*	
S11	0.73910 (10)	0.36381 (8)	0.70722 (5)	0.02472 (15)	
C12	0.5672 (4)	0.3334 (3)	0.62337 (18)	0.0201 (5)	
C13	0.3321 (4)	0.3826 (3)	0.64308 (19)	0.0237 (5)	
H13	0.2105	0.3752	0.6051	0.028*	
C14	0.2892 (4)	0.4455 (3)	0.72571 (19)	0.0257 (5)	
H14	0.1361	0.4855	0.7496	0.031*	
C15	0.4928 (4)	0.4419 (3)	0.76705 (18)	0.0226 (5)	
Br15	0.52162 (5)	0.51602 (4)	0.87603 (2)	0.03340 (11)	
C31	0.5186 (4)	0.1155 (3)	0.33650 (18)	0.0228 (5)	
C32	0.6500 (4)	0.0257 (3)	0.27336 (19)	0.0260 (5)	
H32	0.8183	−0.0232	0.2839	0.031*	
C33	0.5396 (5)	0.0066 (3)	0.1957 (2)	0.0287 (6)	
H33	0.6335	−0.0542	0.1535	0.034*	
C34	0.2949 (5)	0.0745 (3)	0.17838 (19)	0.0269 (5)	
C35	0.1633 (4)	0.1596 (4)	0.2431 (2)	0.0294 (6)	
H35	−0.0053	0.2054	0.2335	0.035*	
C36	0.2716 (4)	0.1791 (3)	0.32083 (19)	0.0270 (5)	
H36	0.1767	0.2365	0.3641	0.032*	
C37	0.1733 (5)	0.0654 (4)	0.0903 (2)	0.0368 (7)	
H37A	0.2707	−0.0368	0.0703	0.044*	
H37B	0.0168	0.0536	0.1074	0.044*	
C38	0.1372 (6)	0.2197 (4)	0.0072 (2)	0.0462 (8)	
H38A	0.0542	0.2101	−0.0479	0.069*	
H38B	0.2921	0.2290	−0.0119	0.069*	
H38C	0.0414	0.3212	0.0268	0.069*	

### (II) (2E)-1-(5-Bromothiophen-2-yl)-3-(4-ethylphenyl)prop-2-en-1-one Atomic displacement parameters (Å^2^)

**Table:**

	*U*^11^	*U*^22^	*U*^33^	*U*^12^	*U*^13^	*U*^23^
C1	0.0230 (11)	0.0202 (12)	0.0231 (13)	−0.0046 (10)	0.0008 (10)	−0.0051 (10)
O1	0.0224 (9)	0.0440 (12)	0.0324 (11)	−0.0077 (8)	0.0034 (8)	−0.0176 (10)
C2	0.0209 (11)	0.0284 (14)	0.0249 (13)	−0.0083 (10)	0.0031 (10)	−0.0096 (11)
C3	0.0253 (12)	0.0258 (13)	0.0232 (13)	−0.0095 (10)	0.0025 (10)	−0.0070 (11)
S11	0.0193 (3)	0.0315 (4)	0.0256 (3)	−0.0089 (3)	0.0004 (2)	−0.0111 (3)
C12	0.0205 (10)	0.0213 (12)	0.0208 (12)	−0.0090 (9)	0.0006 (9)	−0.0071 (10)
C13	0.0216 (11)	0.0272 (13)	0.0261 (13)	−0.0106 (10)	0.0011 (10)	−0.0107 (11)
C14	0.0216 (11)	0.0295 (14)	0.0293 (14)	−0.0096 (10)	0.0062 (10)	−0.0131 (12)
C15	0.0275 (11)	0.0215 (12)	0.0207 (12)	−0.0095 (10)	0.0039 (10)	−0.0073 (10)
Br15	0.04273 (18)	0.03937 (19)	0.02491 (16)	−0.01791 (13)	0.00177 (11)	−0.01472 (13)
C31	0.0266 (12)	0.0234 (13)	0.0202 (12)	−0.0100 (10)	0.0046 (10)	−0.0074 (10)
C32	0.0278 (12)	0.0226 (13)	0.0275 (14)	−0.0060 (10)	0.0044 (10)	−0.0105 (11)
C33	0.0382 (14)	0.0253 (14)	0.0280 (15)	−0.0127 (11)	0.0095 (11)	−0.0146 (12)
C34	0.0360 (13)	0.0277 (14)	0.0248 (13)	−0.0197 (11)	0.0042 (11)	−0.0088 (11)
C35	0.0262 (12)	0.0346 (15)	0.0329 (15)	−0.0138 (11)	0.0044 (11)	−0.0140 (13)
C36	0.0271 (12)	0.0309 (14)	0.0271 (14)	−0.0113 (11)	0.0070 (10)	−0.0136 (12)
C37	0.0421 (15)	0.0477 (19)	0.0337 (16)	−0.0244 (14)	0.0052 (13)	−0.0212 (15)
C38	0.0543 (19)	0.048 (2)	0.0350 (18)	−0.0118 (16)	−0.0089 (15)	−0.0155 (16)

### (II) (2E)-1-(5-Bromothiophen-2-yl)-3-(4-ethylphenyl)prop-2-en-1-one Geometric parameters (Å, º)

**Table:**

C1—O1	1.226 (3)	C31—C32	1.397 (3)
C1—C12	1.464 (3)	C32—C33	1.383 (3)
C1—C2	1.468 (3)	C32—H32	0.9500
C2—C3	1.328 (3)	C33—C34	1.383 (4)
C2—H2	0.9500	C33—H33	0.9500
C3—C31	1.461 (3)	C34—C35	1.394 (3)
C3—H3	0.9500	C34—C37	1.507 (3)
S11—C15	1.705 (2)	C35—C36	1.379 (3)
S11—C12	1.729 (2)	C35—H35	0.9500
C12—C13	1.367 (3)	C36—H36	0.9500
C13—C14	1.409 (3)	C37—C38	1.494 (5)
C13—H13	0.9500	C37—H37A	0.9900
C14—C15	1.358 (3)	C37—H37B	0.9900
C14—H14	0.9500	C38—H38A	0.9800
C15—Br15	1.868 (2)	C38—H38B	0.9800
C31—C36	1.393 (3)	C38—H38C	0.9800
			
O1—C1—C12	119.6 (2)	C33—C32—H32	119.4
O1—C1—C2	123.1 (2)	C31—C32—H32	119.4
C12—C1—C2	117.3 (2)	C34—C33—C32	121.2 (2)
C3—C2—C1	122.0 (2)	C34—C33—H33	119.4
C3—C2—H2	119.0	C32—C33—H33	119.4
C1—C2—H2	119.0	C33—C34—C35	117.6 (2)
C2—C3—C31	127.2 (2)	C33—C34—C37	121.8 (2)
C2—C3—H3	116.4	C35—C34—C37	120.5 (2)
C31—C3—H3	116.4	C36—C35—C34	121.7 (2)
C15—S11—C12	90.81 (11)	C36—C35—H35	119.2
C13—C12—C1	131.3 (2)	C34—C35—H35	119.2
C13—C12—S11	111.33 (17)	C35—C36—C31	120.7 (2)
C1—C12—S11	117.35 (16)	C35—C36—H36	119.6
C12—C13—C14	112.9 (2)	C31—C36—H36	119.6
C12—C13—H13	123.6	C38—C37—C34	112.1 (2)
C14—C13—H13	123.6	C38—C37—H37A	109.2
C15—C14—C13	111.9 (2)	C34—C37—H37A	109.2
C15—C14—H14	124.1	C38—C37—H37B	109.2
C13—C14—H14	124.1	C34—C37—H37B	109.2
C14—C15—S11	113.11 (17)	H37A—C37—H37B	107.9
C14—C15—Br15	126.95 (18)	C37—C38—H38A	109.5
S11—C15—Br15	119.93 (13)	C37—C38—H38B	109.5
C36—C31—C32	117.6 (2)	H38A—C38—H38B	109.5
C36—C31—C3	122.8 (2)	C37—C38—H38C	109.5
C32—C31—C3	119.5 (2)	H38A—C38—H38C	109.5
C33—C32—C31	121.2 (2)	H38B—C38—H38C	109.5
			
O1—C1—C2—C3	7.6 (4)	C12—S11—C15—Br15	−178.81 (16)
C12—C1—C2—C3	−173.3 (3)	C2—C3—C31—C36	3.5 (4)
C1—C2—C3—C31	−175.6 (2)	C2—C3—C31—C32	−179.5 (3)
O1—C1—C12—C13	177.6 (3)	C36—C31—C32—C33	2.6 (4)
C2—C1—C12—C13	−1.5 (4)	C3—C31—C32—C33	−174.6 (2)
O1—C1—C12—S11	−3.5 (3)	C31—C32—C33—C34	−0.6 (4)
C2—C1—C12—S11	177.34 (18)	C32—C33—C34—C35	−1.3 (4)
C15—S11—C12—C13	0.0 (2)	C32—C33—C34—C37	175.7 (2)
C15—S11—C12—C1	−179.1 (2)	C33—C34—C35—C36	1.2 (4)
C1—C12—C13—C14	179.0 (3)	C37—C34—C35—C36	−175.8 (3)
S11—C12—C13—C14	0.1 (3)	C34—C35—C36—C31	0.8 (4)
C12—C13—C14—C15	−0.2 (3)	C32—C31—C36—C35	−2.7 (4)
C13—C14—C15—S11	0.2 (3)	C3—C31—C36—C35	174.4 (2)
C13—C14—C15—Br15	178.79 (19)	C33—C34—C37—C38	−92.6 (3)
C12—S11—C15—C14	−0.1 (2)	C35—C34—C37—C38	84.4 (3)

### (III) (2E)-1-(5-Chlorothiophen-2-yl)-3-(4-ethoxyphenyl)prop-2-en-1-one Crystal data

**Table:**

C_15_H_13_ClO_2_S	*F*(000) = 608
*M**_r_* = 292.76	*D*_x_ = 1.440 Mg m^−^^3^
Monoclinic, *P*2_1_/*c*	Mo *K*α radiation, λ = 0.71073 Å
*a* = 16.3577 (6) Å	Cell parameters from 4472 reflections
*b* = 7.4518 (4) Å	θ = 3.6–32.8°
*c* = 11.0892 (4) Å	µ = 0.43 mm^−^^1^
β = 92.260 (3)°	*T* = 173 K
*V* = 1350.66 (10) Å^3^	Needle, colourless
*Z* = 4	0.50 × 0.28 × 0.17 mm

### (III) (2E)-1-(5-Chlorothiophen-2-yl)-3-(4-ethoxyphenyl)prop-2-en-1-one Data collection

**Table:**

Agilent Eos Gemini diffractometer	3102 reflections with *I* > 2σ(*I*)
Radiation source: Enhance (Mo) X-ray Source	*R*_int_ = 0.038
ω scans	θ_max_ = 30.0°, θ_min_ = 3.6°
Absorption correction: multi-scan (CrysAlis RED; Agilent, 2012)	*h* = −23→12
*T*_min_ = 0.789, *T*_max_ = 0.929	*k* = −9→10
7925 measured reflections	*l* = −15→15
3933 independent reflections	

### (III) (2E)-1-(5-Chlorothiophen-2-yl)-3-(4-ethoxyphenyl)prop-2-en-1-one Refinement

**Table:**

Refinement on *F*^2^	0 restraints
Least-squares matrix: full	Hydrogen site location: inferred from neighbouring sites
*R*[*F*^2^ > 2σ(*F*^2^)] = 0.041	H-atom parameters constrained
*wR*(*F*^2^) = 0.111	*w* = 1/[σ^2^(*F*_o_^2^) + (0.0511*P*)^2^] where *P* = (*F*_o_^2^ + 2*F*_c_^2^)/3
*S* = 1.06	(Δ/σ)_max_ < 0.001
3933 reflections	Δρ_max_ = 0.28 e Å^−^^3^
173 parameters	Δρ_min_ = −0.39 e Å^−^^3^

### (III) (2E)-1-(5-Chlorothiophen-2-yl)-3-(4-ethoxyphenyl)prop-2-en-1-one Special details

**Table:**

Geometry. All e.s.d.'s (except the e.s.d. in the dihedral angle between two l.s. planes) are estimated using the full covariance matrix. The cell e.s.d.'s are taken into account individually in the estimation of e.s.d.'s in distances, angles and torsion angles; correlations between e.s.d.'s in cell parameters are only used when they are defined by crystal symmetry. An approximate (isotropic) treatment of cell e.s.d.'s is used for estimating e.s.d.'s involving l.s. planes.

### (III) (2E)-1-(5-Chlorothiophen-2-yl)-3-(4-ethoxyphenyl)prop-2-en-1-one Fractional atomic coordinates and isotropic or equivalent isotropic displacement parameters (Å^2^)

**Table:**

	*x*	*y*	*z*	*U*_iso_*/*U*_eq_	
C1	0.48158 (9)	0.8068 (2)	0.36984 (12)	0.0216 (3)	
O1	0.46622 (7)	0.8563 (2)	0.26556 (9)	0.0323 (3)	
C2	0.42015 (9)	0.7307 (2)	0.44768 (12)	0.0224 (3)	
H2	0.4360	0.6950	0.5275	0.027*	
C3	0.34250 (9)	0.7104 (2)	0.40898 (12)	0.0203 (3)	
H3	0.3296	0.7470	0.3284	0.024*	
S11	0.63794 (2)	0.91918 (6)	0.33294 (3)	0.02406 (12)	
C12	0.56528 (9)	0.8246 (2)	0.42167 (12)	0.0200 (3)	
C13	0.59796 (9)	0.7742 (2)	0.53208 (13)	0.0219 (3)	
H313	0.5670	0.7205	0.5932	0.026*	
C14	0.68245 (9)	0.8104 (2)	0.54546 (13)	0.0233 (3)	
H14	0.7151	0.7840	0.6160	0.028*	
C15	0.71118 (9)	0.8880 (2)	0.44464 (13)	0.0217 (3)	
Cl15	0.81004 (2)	0.95089 (6)	0.42162 (4)	0.03018 (12)	
C31	0.27552 (9)	0.6388 (2)	0.47614 (12)	0.0189 (3)	
C32	0.19780 (9)	0.6176 (2)	0.41992 (13)	0.0229 (3)	
H32	0.1900	0.6488	0.3372	0.027*	
C33	0.13266 (9)	0.5529 (2)	0.48145 (14)	0.0245 (3)	
H33	0.0807	0.5394	0.4412	0.029*	
C34	0.14307 (9)	0.5074 (2)	0.60284 (13)	0.0215 (3)	
C35	0.21977 (9)	0.5257 (2)	0.66077 (13)	0.0220 (3)	
H35	0.2274	0.4940	0.7434	0.026*	
C36	0.28472 (9)	0.5902 (2)	0.59763 (13)	0.0212 (3)	
H36	0.3369	0.6017	0.6377	0.025*	
O34	0.07523 (7)	0.44649 (17)	0.65798 (10)	0.0274 (3)	
C37	0.08278 (10)	0.4097 (3)	0.78481 (14)	0.0304 (4)	
H37A	0.1087	0.5125	0.8281	0.036*	
H37B	0.1170	0.3019	0.7999	0.036*	
C38	−0.00225 (12)	0.3790 (3)	0.82785 (18)	0.0427 (5)	
H38A	−0.0281	0.2806	0.7817	0.064*	
H38B	−0.0347	0.4886	0.8161	0.064*	
H38C	0.0007	0.3476	0.9137	0.064*	

### (III) (2E)-1-(5-Chlorothiophen-2-yl)-3-(4-ethoxyphenyl)prop-2-en-1-one Atomic displacement parameters (Å^2^)

**Table:**

	*U*^11^	*U*^22^	*U*^33^	*U*^12^	*U*^13^	*U*^23^
C1	0.0198 (7)	0.0260 (8)	0.0190 (6)	0.0008 (7)	0.0008 (5)	−0.0012 (6)
O1	0.0234 (6)	0.0528 (9)	0.0206 (5)	−0.0050 (6)	−0.0009 (4)	0.0065 (5)
C2	0.0215 (7)	0.0261 (8)	0.0196 (6)	−0.0009 (7)	0.0016 (5)	0.0003 (6)
C3	0.0214 (7)	0.0216 (8)	0.0180 (6)	0.0004 (6)	0.0018 (5)	−0.0024 (5)
S11	0.02107 (19)	0.0335 (2)	0.01778 (18)	−0.00399 (17)	0.00259 (14)	0.00242 (15)
C12	0.0185 (7)	0.0230 (8)	0.0185 (6)	−0.0016 (6)	0.0033 (5)	−0.0008 (5)
C13	0.0212 (7)	0.0255 (8)	0.0192 (6)	−0.0018 (7)	0.0026 (5)	0.0020 (6)
C14	0.0216 (7)	0.0250 (8)	0.0231 (7)	−0.0011 (7)	−0.0022 (5)	0.0013 (6)
C15	0.0185 (7)	0.0209 (8)	0.0258 (7)	−0.0007 (6)	0.0014 (6)	−0.0041 (6)
Cl15	0.01899 (19)	0.0340 (3)	0.0378 (2)	−0.00428 (17)	0.00436 (16)	−0.00219 (17)
C31	0.0175 (6)	0.0190 (8)	0.0202 (6)	0.0008 (6)	0.0009 (5)	−0.0031 (6)
C32	0.0214 (7)	0.0276 (9)	0.0195 (7)	0.0005 (7)	−0.0018 (5)	−0.0001 (6)
C33	0.0176 (7)	0.0297 (9)	0.0260 (7)	−0.0006 (7)	−0.0040 (6)	−0.0026 (6)
C34	0.0172 (7)	0.0217 (8)	0.0257 (7)	−0.0007 (6)	0.0032 (6)	−0.0024 (6)
C35	0.0202 (7)	0.0269 (8)	0.0188 (7)	0.0010 (7)	0.0008 (5)	−0.0003 (6)
C36	0.0174 (7)	0.0247 (8)	0.0213 (7)	−0.0003 (6)	−0.0021 (5)	−0.0021 (6)
O34	0.0190 (5)	0.0351 (7)	0.0283 (6)	−0.0048 (5)	0.0039 (4)	0.0023 (5)
C37	0.0279 (8)	0.0358 (10)	0.0279 (8)	−0.0025 (8)	0.0072 (7)	0.0027 (7)
C38	0.0357 (10)	0.0494 (13)	0.0440 (10)	−0.0071 (10)	0.0159 (8)	0.0049 (9)

### (III) (2E)-1-(5-Chlorothiophen-2-yl)-3-(4-ethoxyphenyl)prop-2-en-1-one Geometric parameters (Å, º)

**Table:**

C1—O1	1.2302 (17)	C32—C33	1.375 (2)
C1—C2	1.465 (2)	C32—H32	0.9500
C1—C12	1.4698 (19)	C33—C34	1.392 (2)
C2—C3	1.333 (2)	C33—H33	0.9500
C2—H2	0.9500	C34—O34	1.3654 (19)
C3—C31	1.451 (2)	C34—C35	1.394 (2)
C3—H3	0.9500	C35—C36	1.381 (2)
S11—C15	1.7049 (15)	C35—H35	0.9500
S11—C12	1.7233 (15)	C36—H36	0.9500
C12—C13	1.369 (2)	O34—C37	1.4335 (19)
C13—C14	1.410 (2)	C37—C38	1.506 (2)
C13—H313	0.9500	C37—H37A	0.9900
C14—C15	1.359 (2)	C37—H37B	0.9900
C14—H14	0.9500	C38—H38A	0.9800
C15—Cl15	1.7125 (15)	C38—H38B	0.9800
C31—C36	1.398 (2)	C38—H38C	0.9800
C31—C32	1.4027 (19)		
			
O1—C1—C2	123.41 (13)	C31—C32—H32	119.2
O1—C1—C12	119.55 (13)	C32—C33—C34	119.86 (14)
C2—C1—C12	117.04 (12)	C32—C33—H33	120.1
C3—C2—C1	121.42 (13)	C34—C33—H33	120.1
C3—C2—H2	119.3	O34—C34—C33	116.33 (13)
C1—C2—H2	119.3	O34—C34—C35	123.98 (13)
C2—C3—C31	127.28 (13)	C33—C34—C35	119.69 (14)
C2—C3—H3	116.4	C36—C35—C34	119.79 (14)
C31—C3—H3	116.4	C36—C35—H35	120.1
C15—S11—C12	90.58 (7)	C34—C35—H35	120.1
C13—C12—C1	130.54 (14)	C35—C36—C31	121.54 (14)
C13—C12—S11	111.66 (11)	C35—C36—H36	119.2
C1—C12—S11	117.79 (10)	C31—C36—H36	119.2
C12—C13—C14	112.83 (13)	C34—O34—C37	117.57 (12)
C12—C13—H313	123.6	O34—C37—C38	107.17 (14)
C14—C13—H313	123.6	O34—C37—H37A	110.3
C15—C14—C13	111.36 (13)	C38—C37—H37A	110.3
C15—C14—H14	124.3	O34—C37—H37B	110.3
C13—C14—H14	124.3	C38—C37—H37B	110.3
C14—C15—S11	113.57 (11)	H37A—C37—H37B	108.5
C14—C15—Cl15	126.75 (12)	C37—C38—H38A	109.5
S11—C15—Cl15	119.67 (9)	C37—C38—H38B	109.5
C36—C31—C32	117.42 (13)	H38A—C38—H38B	109.5
C36—C31—C3	122.39 (13)	C37—C38—H38C	109.5
C32—C31—C3	120.20 (12)	H38A—C38—H38C	109.5
C33—C32—C31	121.70 (13)	H38B—C38—H38C	109.5
C33—C32—H32	119.2		
			
O1—C1—C2—C3	0.0 (3)	C2—C3—C31—C36	4.0 (3)
C12—C1—C2—C3	179.55 (15)	C2—C3—C31—C32	−176.64 (16)
C1—C2—C3—C31	−179.46 (15)	C36—C31—C32—C33	0.5 (2)
O1—C1—C12—C13	−177.31 (17)	C3—C31—C32—C33	−178.92 (15)
C2—C1—C12—C13	3.1 (3)	C31—C32—C33—C34	0.2 (3)
O1—C1—C12—S11	1.2 (2)	C32—C33—C34—O34	179.12 (15)
C2—C1—C12—S11	−178.36 (12)	C32—C33—C34—C35	−0.7 (2)
C15—S11—C12—C13	0.49 (13)	O34—C34—C35—C36	−179.38 (15)
C15—S11—C12—C1	−178.32 (13)	C33—C34—C35—C36	0.5 (2)
C1—C12—C13—C14	178.12 (16)	C34—C35—C36—C31	0.3 (2)
S11—C12—C13—C14	−0.50 (18)	C32—C31—C36—C35	−0.8 (2)
C12—C13—C14—C15	0.2 (2)	C3—C31—C36—C35	178.63 (15)
C13—C14—C15—S11	0.15 (19)	C33—C34—O34—C37	−175.99 (15)
C13—C14—C15—Cl15	−179.02 (12)	C35—C34—O34—C37	3.9 (2)
C12—S11—C15—C14	−0.37 (14)	C34—O34—C37—C38	169.60 (16)
C12—S11—C15—Cl15	178.87 (11)		

### (III) (2E)-1-(5-Chlorothiophen-2-yl)-3-(4-ethoxyphenyl)prop-2-en-1-one Hydrogen-bond geometry (Å, º)

**Table:**

*D*—H···*A*	*D*—H	H···*A*	*D*···*A*	*D*—H···*A*
C36—H36···O1^i^	0.95	2.52	3.4649 (18)	173

Symmetry code: (i) *x*, −*y*+3/2, *z*+1/2.

### (IV) (2E)-1-(5-Bromothiophen-2-yl)-3-(4-ethoxyphenyl)prop-2-en-1-one Crystal data

**Table:**

C_15_H_13_BrO_2_S	*F*(000) = 680
*M**_r_* = 337.21	*D*_x_ = 1.617 Mg m^−^^3^
Monoclinic, *P*2_1_/*c*	Mo *K*α radiation, λ = 0.71073 Å
*a* = 16.5498 (7) Å	Cell parameters from 4590 reflections
*b* = 7.5069 (4) Å	θ = 3.3–32.8°
*c* = 11.1574 (5) Å	µ = 3.11 mm^−^^1^
β = 92.618 (4)°	*T* = 173 K
*V* = 1384.72 (11) Å^3^	Needle, colourless
*Z* = 4	0.58 × 0.32 × 0.29 mm

### (IV) (2E)-1-(5-Bromothiophen-2-yl)-3-(4-ethoxyphenyl)prop-2-en-1-one Data collection

**Table:**

Agilent Eos Gemini diffractometer	3189 reflections with *I* > 2σ(*I*)
Radiation source: Enhance (Mo) X-ray Source	*R*_int_ = 0.037
ω scans	θ_max_ = 30.0°, θ_min_ = 3.3°
Absorption correction: multi-scan (CrysAlis RED; Agilent, 2012)	*h* = −23→23
*T*_min_ = 0.261, *T*_max_ = 0.405	*k* = −10→7
8866 measured reflections	*l* = −15→10
4040 independent reflections	

### (IV) (2E)-1-(5-Bromothiophen-2-yl)-3-(4-ethoxyphenyl)prop-2-en-1-one Refinement

**Table:**

Refinement on *F*^2^	Hydrogen site location: inferred from neighbouring sites
Least-squares matrix: full	H-atom parameters constrained
*R*[*F*^2^ > 2σ(*F*^2^)] = 0.038	*w* = 1/[σ^2^(*F*_o_^2^) + (0.027*P*)^2^] where *P* = (*F*_o_^2^ + 2*F*_c_^2^)/3
*wR*(*F*^2^) = 0.078	(Δ/σ)_max_ = 0.002
*S* = 1.03	Δρ_max_ = 0.54 e Å^−^^3^
4040 reflections	Δρ_min_ = −0.43 e Å^−^^3^
174 parameters	Extinction correction: SHELXL, Fc^*^=kFc[1+0.001xFc^2^λ^3^/sin(2θ)]^-1/4^
0 restraints	Extinction coefficient: 0.0142 (6)

### (IV) (2E)-1-(5-Bromothiophen-2-yl)-3-(4-ethoxyphenyl)prop-2-en-1-one Special details

**Table:**

Geometry. All e.s.d.'s (except the e.s.d. in the dihedral angle between two l.s. planes) are estimated using the full covariance matrix. The cell e.s.d.'s are taken into account individually in the estimation of e.s.d.'s in distances, angles and torsion angles; correlations between e.s.d.'s in cell parameters are only used when they are defined by crystal symmetry. An approximate (isotropic) treatment of cell e.s.d.'s is used for estimating e.s.d.'s involving l.s. planes.

### (IV) (2E)-1-(5-Bromothiophen-2-yl)-3-(4-ethoxyphenyl)prop-2-en-1-one Fractional atomic coordinates and isotropic or equivalent isotropic displacement parameters (Å^2^)

**Table:**

	*x*	*y*	*z*	*U*_iso_*/*U*_eq_	
C1	0.47683 (13)	0.8021 (3)	0.37094 (19)	0.0214 (5)	
O1	0.46163 (10)	0.8474 (2)	0.26618 (14)	0.0325 (4)	
C2	0.41612 (13)	0.7286 (3)	0.44882 (19)	0.0219 (5)	
H2	0.4316	0.6977	0.5292	0.026*	
C3	0.33987 (13)	0.7040 (3)	0.40968 (19)	0.0198 (4)	
H3	0.3274	0.7351	0.3284	0.024*	
S11	0.63142 (3)	0.91205 (8)	0.33350 (5)	0.02358 (14)	
C12	0.55978 (13)	0.8208 (3)	0.42271 (19)	0.0197 (4)	
C13	0.59275 (14)	0.7744 (3)	0.5328 (2)	0.0237 (5)	
H313	0.5625	0.7231	0.5943	0.028*	
C14	0.67601 (13)	0.8103 (3)	0.5458 (2)	0.0227 (5)	
H14	0.7083	0.7865	0.6165	0.027*	
C15	0.70441 (13)	0.8830 (3)	0.44500 (19)	0.0197 (4)	
Br15	0.81072 (2)	0.95080 (3)	0.41891 (2)	0.02718 (9)	
C31	0.27341 (12)	0.6353 (3)	0.47671 (18)	0.0174 (4)	
C32	0.19658 (13)	0.6155 (3)	0.42083 (19)	0.0220 (5)	
H32	0.1888	0.6455	0.3384	0.026*	
C33	0.13207 (14)	0.5537 (3)	0.4821 (2)	0.0228 (5)	
H33	0.0806	0.5404	0.4420	0.027*	
C34	0.14246 (13)	0.5107 (3)	0.6032 (2)	0.0201 (4)	
C35	0.21844 (14)	0.5284 (3)	0.6608 (2)	0.0214 (5)	
H35	0.2259	0.4987	0.7433	0.026*	
C36	0.28283 (13)	0.5889 (3)	0.59792 (19)	0.0195 (4)	
H36	0.3346	0.5993	0.6378	0.023*	
O34	0.07528 (9)	0.4517 (2)	0.65781 (15)	0.0260 (4)	
C37	0.08296 (15)	0.4163 (3)	0.7841 (2)	0.0294 (5)	
H37A	0.1074	0.5198	0.8270	0.035*	
H37B	0.1180	0.3111	0.7997	0.035*	
C38	−0.00045 (16)	0.3818 (4)	0.8261 (3)	0.0414 (7)	
H38A	−0.0259	0.2861	0.7780	0.062*	
H38B	−0.0330	0.4904	0.8172	0.062*	
H38C	0.0031	0.3462	0.9107	0.062*	

### (IV) (2E)-1-(5-Bromothiophen-2-yl)-3-(4-ethoxyphenyl)prop-2-en-1-one Atomic displacement parameters (Å^2^)

**Table:**

	*U*^11^	*U*^22^	*U*^33^	*U*^12^	*U*^13^	*U*^23^
C1	0.0219 (11)	0.0234 (11)	0.0191 (11)	−0.0007 (9)	0.0017 (8)	−0.0020 (9)
O1	0.0238 (9)	0.0546 (11)	0.0189 (8)	−0.0044 (8)	−0.0014 (7)	0.0074 (8)
C2	0.0217 (11)	0.0276 (12)	0.0164 (11)	−0.0005 (9)	0.0019 (8)	0.0022 (9)
C3	0.0230 (11)	0.0200 (10)	0.0164 (10)	0.0005 (9)	0.0033 (8)	−0.0013 (9)
S11	0.0220 (3)	0.0332 (3)	0.0157 (3)	−0.0040 (2)	0.0026 (2)	0.0027 (2)
C12	0.0191 (11)	0.0228 (11)	0.0173 (10)	−0.0009 (9)	0.0041 (8)	−0.0003 (9)
C13	0.0237 (12)	0.0246 (11)	0.0228 (12)	−0.0026 (10)	0.0019 (9)	0.0039 (10)
C14	0.0225 (11)	0.0236 (11)	0.0216 (11)	−0.0020 (9)	−0.0033 (9)	0.0020 (9)
C15	0.0169 (10)	0.0189 (10)	0.0233 (11)	0.0011 (9)	0.0015 (8)	−0.0016 (9)
Br15	0.01970 (13)	0.02854 (14)	0.03363 (15)	−0.00331 (9)	0.00473 (9)	−0.00198 (10)
C31	0.0179 (10)	0.0166 (10)	0.0176 (10)	0.0017 (8)	0.0012 (8)	−0.0029 (8)
C32	0.0235 (12)	0.0252 (11)	0.0170 (11)	0.0002 (10)	−0.0024 (9)	−0.0011 (9)
C33	0.0162 (11)	0.0269 (12)	0.0250 (12)	−0.0015 (9)	−0.0024 (9)	−0.0018 (10)
C34	0.0177 (11)	0.0195 (10)	0.0232 (11)	0.0006 (9)	0.0030 (9)	−0.0028 (9)
C35	0.0224 (11)	0.0247 (11)	0.0170 (11)	0.0004 (9)	0.0006 (8)	0.0014 (9)
C36	0.0162 (10)	0.0229 (11)	0.0194 (11)	−0.0002 (9)	−0.0009 (8)	−0.0013 (9)
O34	0.0185 (8)	0.0340 (9)	0.0259 (9)	−0.0048 (7)	0.0041 (6)	0.0010 (7)
C37	0.0293 (13)	0.0333 (13)	0.0261 (12)	−0.0032 (11)	0.0074 (10)	0.0028 (11)
C38	0.0353 (15)	0.0489 (16)	0.0414 (16)	−0.0083 (13)	0.0154 (12)	0.0042 (14)

### (IV) (2E)-1-(5-Bromothiophen-2-yl)-3-(4-ethoxyphenyl)prop-2-en-1-one Geometric parameters (Å, º)

**Table:**

C1—O1	1.232 (3)	C32—C33	1.375 (3)
C1—C2	1.466 (3)	C32—H32	0.9500
C1—C12	1.472 (3)	C33—C34	1.392 (3)
C2—C3	1.329 (3)	C33—H33	0.9500
C2—H2	0.9500	C34—O34	1.366 (3)
C3—C31	1.453 (3)	C34—C35	1.392 (3)
C3—H3	0.9500	C35—C36	1.379 (3)
S11—C15	1.708 (2)	C35—H35	0.9500
S11—C12	1.724 (2)	C36—H36	0.9500
C12—C13	1.366 (3)	O34—C37	1.434 (3)
C13—C14	1.405 (3)	C37—C38	1.501 (3)
C13—H313	0.9500	C37—H37A	0.9900
C14—C15	1.353 (3)	C37—H37B	0.9900
C14—H14	0.9500	C38—H38A	0.9800
C15—Br15	1.867 (2)	C38—H38B	0.9800
C31—C36	1.398 (3)	C38—H38C	0.9800
C31—C32	1.398 (3)		
			
O1—C1—C2	123.4 (2)	C31—C32—H32	119.2
O1—C1—C12	119.5 (2)	C32—C33—C34	119.8 (2)
C2—C1—C12	117.09 (19)	C32—C33—H33	120.1
C3—C2—C1	121.6 (2)	C34—C33—H33	120.1
C3—C2—H2	119.2	O34—C34—C33	116.2 (2)
C1—C2—H2	119.2	O34—C34—C35	124.2 (2)
C2—C3—C31	127.6 (2)	C33—C34—C35	119.7 (2)
C2—C3—H3	116.2	C36—C35—C34	120.0 (2)
C31—C3—H3	116.2	C36—C35—H35	120.0
C15—S11—C12	90.62 (10)	C34—C35—H35	120.0
C13—C12—C1	131.0 (2)	C35—C36—C31	121.3 (2)
C13—C12—S11	111.31 (17)	C35—C36—H36	119.4
C1—C12—S11	117.70 (16)	C31—C36—H36	119.4
C12—C13—C14	113.2 (2)	C34—O34—C37	117.45 (18)
C12—C13—H313	123.4	O34—C37—C38	107.3 (2)
C14—C13—H313	123.4	O34—C37—H37A	110.2
C15—C14—C13	111.6 (2)	C38—C37—H37A	110.2
C15—C14—H14	124.2	O34—C37—H37B	110.2
C13—C14—H14	124.2	C38—C37—H37B	110.2
C14—C15—S11	113.31 (16)	H37A—C37—H37B	108.5
C14—C15—Br15	127.23 (17)	C37—C38—H38A	109.5
S11—C15—Br15	119.46 (12)	C37—C38—H38B	109.5
C36—C31—C32	117.6 (2)	H38A—C38—H38B	109.5
C36—C31—C3	122.2 (2)	C37—C38—H38C	109.5
C32—C31—C3	120.17 (19)	H38A—C38—H38C	109.5
C33—C32—C31	121.7 (2)	H38B—C38—H38C	109.5
C33—C32—H32	119.2		
			
O1—C1—C2—C3	0.8 (4)	C2—C3—C31—C36	1.7 (4)
C12—C1—C2—C3	−178.9 (2)	C2—C3—C31—C32	−179.1 (2)
C1—C2—C3—C31	−179.0 (2)	C36—C31—C32—C33	0.4 (3)
O1—C1—C12—C13	−176.9 (2)	C3—C31—C32—C33	−178.9 (2)
C2—C1—C12—C13	2.9 (4)	C31—C32—C33—C34	0.6 (3)
O1—C1—C12—S11	1.8 (3)	C32—C33—C34—O34	179.5 (2)
C2—C1—C12—S11	−178.48 (16)	C32—C33—C34—C35	−0.9 (3)
C15—S11—C12—C13	0.85 (18)	O34—C34—C35—C36	179.9 (2)
C15—S11—C12—C1	−178.06 (17)	C33—C34—C35—C36	0.3 (3)
C1—C12—C13—C14	178.1 (2)	C34—C35—C36—C31	0.6 (3)
S11—C12—C13—C14	−0.6 (3)	C32—C31—C36—C35	−1.0 (3)
C12—C13—C14—C15	−0.1 (3)	C3—C31—C36—C35	178.3 (2)
C13—C14—C15—S11	0.8 (3)	C33—C34—O34—C37	−176.45 (19)
C13—C14—C15—Br15	−179.32 (16)	C35—C34—O34—C37	4.0 (3)
C12—S11—C15—C14	−0.92 (18)	C34—O34—C37—C38	171.3 (2)
C12—S11—C15—Br15	179.15 (13)		

### (IV) (2E)-1-(5-Bromothiophen-2-yl)-3-(4-ethoxyphenyl)prop-2-en-1-one Hydrogen-bond geometry (Å, º)

**Table:**

*D*—H···*A*	*D*—H	H···*A*	*D*···*A*	*D*—H···*A*
C36—H36···O1^i^	0.95	2.52	3.464 (2)	172

Symmetry code: (i) *x*, −*y*+3/2, *z*+1/2.

### (V) (2E)-3-(4-Bromophenyl)-1-(5-chlorothiophen-2-yl)prop-2-en-1-one Crystal data

**Table:**

C_13_H_8_BrClOS	*Z* = 2
*M**_r_* = 327.60	*F*(000) = 324
Triclinic, *P*1	*D*_x_ = 1.763 Mg m^−^^3^
*a* = 6.0152 (8) Å	Mo *K*α radiation, λ = 0.71073 Å
*b* = 8.5691 (12) Å	Cell parameters from 4058 reflections
*c* = 13.1824 (9) Å	θ = 3.2–32.8°
α = 75.25 (1)°	µ = 3.69 mm^−^^1^
β = 81.446 (8)°	*T* = 173 K
γ = 70.281 (12)°	Needle, colourless
*V* = 617.09 (14) Å^3^	0.41 × 0.20 × 0.18 mm

### (V) (2E)-3-(4-Bromophenyl)-1-(5-chlorothiophen-2-yl)prop-2-en-1-one Data collection

**Table:**

Agilent Eos Gemini diffractometer	2817 reflections with *I* > 2σ(*I*)
Radiation source: Enhance (Mo) X-ray Source	*R*_int_ = 0.026
ω scans	θ_max_ = 30.0°, θ_min_ = 3.2°
Absorption correction: multi-scan (CrysAlis RED; Agilent, 2012)	*h* = −8→8
*T*_min_ = 0.298, *T*_max_ = 0.514	*k* = −10→12
6674 measured reflections	*l* = −18→18
3599 independent reflections	

### (V) (2E)-3-(4-Bromophenyl)-1-(5-chlorothiophen-2-yl)prop-2-en-1-one Refinement

**Table:**

Refinement on *F*^2^	0 restraints
Least-squares matrix: full	Hydrogen site location: inferred from neighbouring sites
*R*[*F*^2^ > 2σ(*F*^2^)] = 0.042	H-atom parameters constrained
*wR*(*F*^2^) = 0.100	*w* = 1/[σ^2^(*F*_o_^2^) + (0.0428*P*)^2^ + 0.3839*P*] where *P* = (*F*_o_^2^ + 2*F*_c_^2^)/3
*S* = 1.03	(Δ/σ)_max_ < 0.001
3599 reflections	Δρ_max_ = 1.43 e Å^−^^3^
154 parameters	Δρ_min_ = −0.53 e Å^−^^3^

### (V) (2E)-3-(4-Bromophenyl)-1-(5-chlorothiophen-2-yl)prop-2-en-1-one Special details

**Table:**

Geometry. All e.s.d.'s (except the e.s.d. in the dihedral angle between two l.s. planes) are estimated using the full covariance matrix. The cell e.s.d.'s are taken into account individually in the estimation of e.s.d.'s in distances, angles and torsion angles; correlations between e.s.d.'s in cell parameters are only used when they are defined by crystal symmetry. An approximate (isotropic) treatment of cell e.s.d.'s is used for estimating e.s.d.'s involving l.s. planes.

### (V) (2E)-3-(4-Bromophenyl)-1-(5-chlorothiophen-2-yl)prop-2-en-1-one Fractional atomic coordinates and isotropic or equivalent isotropic displacement parameters (Å^2^)

**Table:**

	*x*	*y*	*z*	*U*_iso_*/*U*_eq_	
C1	0.3079 (5)	0.7031 (4)	0.5481 (2)	0.0219 (5)	
O1	0.0931 (3)	0.7382 (3)	0.54196 (16)	0.0316 (5)	
C2	0.4552 (5)	0.7932 (4)	0.4740 (2)	0.0233 (5)	
H2	0.6221	0.7524	0.4781	0.028*	
C3	0.3594 (5)	0.9316 (4)	0.4008 (2)	0.0235 (5)	
H3	0.1914	0.9731	0.4024	0.028*	
S11	0.25627 (12)	0.46020 (9)	0.72271 (5)	0.02442 (15)	
C12	0.4287 (5)	0.5637 (3)	0.63199 (19)	0.0209 (5)	
C13	0.6614 (5)	0.4996 (4)	0.6548 (2)	0.0230 (5)	
H13	0.7821	0.5412	0.6141	0.028*	
C14	0.7041 (5)	0.3649 (4)	0.7453 (2)	0.0234 (5)	
H14	0.8555	0.3055	0.7719	0.028*	
C15	0.5014 (5)	0.3316 (3)	0.7890 (2)	0.0228 (5)	
Cl15	0.47267 (14)	0.18017 (10)	0.89894 (5)	0.03458 (18)	
C31	0.4883 (5)	1.0255 (3)	0.3183 (2)	0.0215 (5)	
C32	0.3621 (5)	1.1691 (3)	0.2476 (2)	0.0236 (5)	
H32	0.1943	1.2092	0.2575	0.028*	
C33	0.4754 (5)	1.2549 (4)	0.1632 (2)	0.0250 (5)	
H33	0.3868	1.3509	0.1151	0.030*	
C34	0.7182 (5)	1.1974 (4)	0.1513 (2)	0.0247 (6)	
Br34	0.87986 (6)	1.31203 (4)	0.03812 (2)	0.03776 (12)	
C35	0.8519 (5)	1.0562 (4)	0.2200 (2)	0.0268 (6)	
H35	1.0197	1.0177	0.2099	0.032*	
C36	0.7356 (5)	0.9725 (4)	0.3037 (2)	0.0255 (6)	
H36	0.8255	0.8775	0.3519	0.031*	

### (V) (2E)-3-(4-Bromophenyl)-1-(5-chlorothiophen-2-yl)prop-2-en-1-one Atomic displacement parameters (Å^2^)

**Table:**

	*U*^11^	*U*^22^	*U*^33^	*U*^12^	*U*^13^	*U*^23^
C1	0.0238 (13)	0.0213 (13)	0.0195 (12)	−0.0059 (10)	−0.0023 (10)	−0.0036 (10)
O1	0.0206 (10)	0.0337 (12)	0.0348 (11)	−0.0058 (9)	−0.0044 (8)	0.0004 (9)
C2	0.0221 (12)	0.0250 (14)	0.0221 (12)	−0.0089 (11)	−0.0016 (10)	−0.0022 (10)
C3	0.0238 (13)	0.0227 (14)	0.0232 (12)	−0.0067 (11)	−0.0019 (10)	−0.0043 (10)
S11	0.0193 (3)	0.0278 (4)	0.0247 (3)	−0.0094 (3)	0.0003 (2)	−0.0015 (3)
C12	0.0232 (13)	0.0203 (13)	0.0196 (11)	−0.0090 (10)	−0.0011 (10)	−0.0023 (9)
C13	0.0223 (12)	0.0255 (14)	0.0226 (12)	−0.0108 (11)	−0.0015 (10)	−0.0031 (10)
C14	0.0216 (12)	0.0241 (14)	0.0231 (12)	−0.0054 (11)	−0.0050 (10)	−0.0029 (10)
C15	0.0259 (13)	0.0216 (13)	0.0206 (12)	−0.0084 (11)	−0.0022 (10)	−0.0025 (10)
Cl15	0.0439 (4)	0.0324 (4)	0.0251 (3)	−0.0175 (3)	−0.0011 (3)	0.0046 (3)
C31	0.0228 (13)	0.0213 (13)	0.0206 (12)	−0.0070 (10)	−0.0027 (10)	−0.0043 (10)
C32	0.0227 (13)	0.0203 (14)	0.0237 (12)	−0.0013 (10)	−0.0050 (10)	−0.0035 (10)
C33	0.0279 (14)	0.0201 (13)	0.0242 (13)	−0.0045 (11)	−0.0082 (11)	−0.0005 (10)
C34	0.0307 (14)	0.0243 (14)	0.0206 (12)	−0.0133 (12)	−0.0031 (10)	−0.0005 (10)
Br34	0.03904 (19)	0.0438 (2)	0.02867 (17)	−0.02171 (15)	−0.00236 (12)	0.00715 (13)
C35	0.0224 (13)	0.0262 (15)	0.0302 (14)	−0.0080 (11)	−0.0060 (11)	−0.0005 (11)
C36	0.0217 (13)	0.0247 (14)	0.0259 (13)	−0.0054 (11)	−0.0061 (10)	0.0018 (10)

### (V) (2E)-3-(4-Bromophenyl)-1-(5-chlorothiophen-2-yl)prop-2-en-1-one Geometric parameters (Å, º)

**Table:**

C1—O1	1.234 (3)	C14—H14	0.9500
C1—C12	1.469 (4)	C15—Cl15	1.712 (3)
C1—C2	1.469 (4)	C31—C32	1.398 (4)
C2—C3	1.338 (4)	C31—C36	1.400 (4)
C2—H2	0.9500	C32—C33	1.393 (4)
C3—C31	1.469 (4)	C32—H32	0.9500
C3—H3	0.9500	C33—C34	1.374 (4)
S11—C15	1.718 (3)	C33—H33	0.9500
S11—C12	1.733 (3)	C34—C35	1.390 (4)
C12—C13	1.369 (4)	C34—Br34	1.898 (3)
C13—C14	1.418 (4)	C35—C36	1.388 (4)
C13—H13	0.9500	C35—H35	0.9500
C14—C15	1.357 (4)	C36—H36	0.9500
			
O1—C1—C12	120.0 (2)	C14—C15—S11	113.4 (2)
O1—C1—C2	123.2 (2)	Cl15—C15—S11	119.76 (16)
C12—C1—C2	116.8 (2)	C32—C31—C36	117.8 (2)
C3—C2—C1	121.4 (2)	C32—C31—C3	119.6 (2)
C3—C2—H2	119.3	C36—C31—C3	122.6 (2)
C1—C2—H2	119.3	C33—C32—C31	121.8 (3)
C2—C3—C31	126.4 (3)	C33—C32—H32	119.1
C2—C3—H3	116.8	C31—C32—H32	119.1
C31—C3—H3	116.8	C34—C33—C32	118.4 (2)
C15—S11—C12	90.62 (13)	C34—C33—H33	120.8
C13—C12—C1	131.2 (2)	C32—C33—H33	120.8
C13—C12—S11	111.4 (2)	C33—C34—C35	122.0 (2)
C1—C12—S11	117.45 (19)	C33—C34—Br34	119.8 (2)
C12—C13—C14	113.2 (2)	C35—C34—Br34	118.3 (2)
C12—C13—H13	123.4	C36—C35—C34	118.7 (3)
C14—C13—H13	123.4	C36—C35—H35	120.6
C15—C14—C13	111.4 (2)	C34—C35—H35	120.6
C15—C14—H14	124.3	C35—C36—C31	121.3 (3)
C13—C14—H14	124.3	C35—C36—H36	119.4
C14—C15—Cl15	126.9 (2)	C31—C36—H36	119.4
			
O1—C1—C2—C3	6.9 (4)	C12—S11—C15—C14	0.1 (2)
C12—C1—C2—C3	−173.8 (3)	C12—S11—C15—Cl15	−179.89 (18)
C1—C2—C3—C31	−175.7 (2)	C2—C3—C31—C32	−179.7 (3)
O1—C1—C12—C13	179.2 (3)	C2—C3—C31—C36	3.6 (4)
C2—C1—C12—C13	−0.1 (4)	C36—C31—C32—C33	1.9 (4)
O1—C1—C12—S11	−2.7 (4)	C3—C31—C32—C33	−175.0 (2)
C2—C1—C12—S11	177.99 (19)	C31—C32—C33—C34	−1.3 (4)
C15—S11—C12—C13	−0.4 (2)	C32—C33—C34—C35	0.7 (4)
C15—S11—C12—C1	−178.8 (2)	C32—C33—C34—Br34	−179.3 (2)
C1—C12—C13—C14	178.7 (3)	C33—C34—C35—C36	−0.7 (4)
S11—C12—C13—C14	0.6 (3)	Br34—C34—C35—C36	179.3 (2)
C12—C13—C14—C15	−0.5 (3)	C34—C35—C36—C31	1.4 (4)
C13—C14—C15—Cl15	−179.8 (2)	C32—C31—C36—C35	−2.0 (4)
C13—C14—C15—S11	0.1 (3)	C3—C31—C36—C35	174.9 (3)

### (VI) (2E)-1-(5-Bromothiophen-2-yl)-3-(3-methoxyphenyl)prop-2-en-1-one Crystal data

**Table:**

C_14_H_11_BrO_2_S	*F*(000) = 648
*M**_r_* = 323.19	*D*_x_ = 1.675 Mg m^−^^3^
Monoclinic, *P*2_1_/*c*	Mo *K*α radiation, λ = 0.71073 Å
*a* = 9.2726 (6) Å	Cell parameters from 4224 reflections
*b* = 11.3948 (8) Å	θ = 3.4–32.7°
*c* = 12.1472 (7) Å	µ = 3.36 mm^−^^1^
β = 93.273 (6)°	*T* = 173 K
*V* = 1281.37 (14) Å^3^	Block, colourless
*Z* = 4	0.54 × 0.42 × 0.31 mm

### (VI) (2E)-1-(5-Bromothiophen-2-yl)-3-(3-methoxyphenyl)prop-2-en-1-one Data collection

**Table:**

Agilent Eos Gemini diffractometer	2914 reflections with *I* > 2σ(*I*)
Radiation source: Enhance (Mo) X-ray Source	*R*_int_ = 0.035
ω scans	θ_max_ = 30.0°, θ_min_ = 3.4°
Absorption correction: multi-scan (CrysAlis RED; Agilent, 2012)	*h* = −13→12
*T*_min_ = 0.216, *T*_max_ = 0.353	*k* = −15→16
8260 measured reflections	*l* = −16→17
3722 independent reflections	

### (VI) (2E)-1-(5-Bromothiophen-2-yl)-3-(3-methoxyphenyl)prop-2-en-1-one Refinement

**Table:**

Refinement on *F*^2^	0 restraints
Least-squares matrix: full	Hydrogen site location: inferred from neighbouring sites
*R*[*F*^2^ > 2σ(*F*^2^)] = 0.034	H-atom parameters constrained
*wR*(*F*^2^) = 0.075	*w* = 1/[σ^2^(*F*_o_^2^) + (0.0293*P*)^2^] where *P* = (*F*_o_^2^ + 2*F*_c_^2^)/3
*S* = 1.02	(Δ/σ)_max_ = 0.003
3722 reflections	Δρ_max_ = 0.49 e Å^−^^3^
164 parameters	Δρ_min_ = −0.46 e Å^−^^3^

### (VI) (2E)-1-(5-Bromothiophen-2-yl)-3-(3-methoxyphenyl)prop-2-en-1-one Special details

**Table:**

Geometry. All e.s.d.'s (except the e.s.d. in the dihedral angle between two l.s. planes) are estimated using the full covariance matrix. The cell e.s.d.'s are taken into account individually in the estimation of e.s.d.'s in distances, angles and torsion angles; correlations between e.s.d.'s in cell parameters are only used when they are defined by crystal symmetry. An approximate (isotropic) treatment of cell e.s.d.'s is used for estimating e.s.d.'s involving l.s. planes.

### (VI) (2E)-1-(5-Bromothiophen-2-yl)-3-(3-methoxyphenyl)prop-2-en-1-one Fractional atomic coordinates and isotropic or equivalent isotropic displacement parameters (Å^2^)

**Table:**

	*x*	*y*	*z*	*U*_iso_*/*U*_eq_	
C1	0.4180 (2)	0.29186 (18)	0.72203 (17)	0.0205 (4)	
O1	0.41175 (17)	0.39954 (13)	0.71617 (13)	0.0310 (4)	
C2	0.5005 (2)	0.22053 (19)	0.64696 (17)	0.0217 (4)	
H2	0.5078	0.1382	0.6584	0.026*	
C3	0.5653 (2)	0.26916 (19)	0.56325 (15)	0.0204 (4)	
H3	0.5545	0.3516	0.5549	0.024*	
S11	0.22557 (6)	0.31118 (4)	0.88317 (4)	0.02124 (12)	
C12	0.3390 (2)	0.23001 (18)	0.80567 (16)	0.0180 (4)	
C13	0.3392 (2)	0.11390 (18)	0.83642 (17)	0.0232 (4)	
H13	0.3952	0.0553	0.8031	0.028*	
C14	0.2474 (2)	0.09085 (19)	0.92288 (17)	0.0245 (5)	
H14	0.2342	0.0155	0.9542	0.029*	
C15	0.1805 (2)	0.18936 (17)	0.95556 (16)	0.0192 (4)	
Br15	0.05413 (2)	0.20408 (2)	1.06918 (2)	0.02654 (8)	
C31	0.6513 (2)	0.20960 (18)	0.48261 (16)	0.0178 (4)	
C32	0.7142 (2)	0.27779 (18)	0.40207 (16)	0.0185 (4)	
H32	0.6980	0.3601	0.3996	0.022*	
C33	0.7994 (2)	0.22577 (18)	0.32643 (16)	0.0192 (4)	
C34	0.8214 (2)	0.10536 (18)	0.32844 (17)	0.0242 (5)	
H34	0.8796	0.0695	0.2761	0.029*	
C35	0.7583 (2)	0.0380 (2)	0.40685 (17)	0.0279 (5)	
H35	0.7730	−0.0445	0.4078	0.034*	
C36	0.6737 (2)	0.08920 (18)	0.48427 (17)	0.0237 (5)	
H36	0.6314	0.0420	0.5382	0.028*	
O33	0.86717 (16)	0.28429 (13)	0.24569 (13)	0.0256 (3)	
C37	0.8641 (3)	0.40869 (19)	0.24766 (19)	0.0309 (5)	
H37A	0.9166	0.4394	0.1861	0.046*	
H37B	0.9099	0.4368	0.3174	0.046*	
H37C	0.7637	0.4358	0.2408	0.046*	

### (VI) (2E)-1-(5-Bromothiophen-2-yl)-3-(3-methoxyphenyl)prop-2-en-1-one Atomic displacement parameters (Å^2^)

**Table:**

	*U*^11^	*U*^22^	*U*^33^	*U*^12^	*U*^13^	*U*^23^
C1	0.0191 (10)	0.0234 (11)	0.0194 (10)	0.0018 (8)	0.0043 (8)	−0.0007 (9)
O1	0.0368 (9)	0.0211 (8)	0.0371 (9)	0.0005 (7)	0.0201 (7)	0.0008 (7)
C2	0.0216 (10)	0.0232 (10)	0.0208 (10)	0.0029 (9)	0.0072 (8)	−0.0020 (9)
C3	0.0200 (10)	0.0211 (10)	0.0204 (10)	0.0034 (8)	0.0038 (8)	−0.0012 (9)
S11	0.0244 (3)	0.0195 (2)	0.0210 (3)	0.0029 (2)	0.0104 (2)	−0.0005 (2)
C12	0.0177 (10)	0.0208 (10)	0.0161 (9)	0.0013 (8)	0.0051 (7)	−0.0045 (8)
C13	0.0277 (11)	0.0197 (10)	0.0229 (11)	0.0033 (9)	0.0071 (8)	−0.0024 (9)
C14	0.0305 (12)	0.0203 (10)	0.0237 (11)	0.0000 (9)	0.0099 (9)	0.0023 (9)
C15	0.0179 (10)	0.0249 (11)	0.0152 (9)	−0.0025 (8)	0.0042 (7)	0.0017 (8)
Br15	0.02519 (12)	0.03558 (14)	0.01992 (12)	0.00135 (9)	0.01063 (8)	0.00304 (9)
C31	0.0153 (9)	0.0249 (10)	0.0133 (9)	−0.0004 (8)	0.0020 (7)	−0.0016 (8)
C32	0.0194 (10)	0.0184 (9)	0.0179 (9)	0.0011 (8)	0.0026 (7)	−0.0005 (8)
C33	0.0175 (10)	0.0233 (10)	0.0169 (9)	0.0017 (8)	0.0029 (7)	0.0029 (9)
C34	0.0296 (11)	0.0234 (10)	0.0206 (10)	0.0044 (9)	0.0100 (8)	−0.0038 (9)
C35	0.0384 (13)	0.0199 (10)	0.0265 (11)	0.0052 (9)	0.0103 (9)	0.0017 (9)
C36	0.0306 (11)	0.0214 (11)	0.0197 (10)	−0.0008 (9)	0.0079 (8)	0.0025 (9)
O33	0.0301 (8)	0.0232 (8)	0.0252 (8)	0.0013 (6)	0.0166 (6)	0.0030 (7)
C37	0.0375 (13)	0.0220 (11)	0.0346 (13)	−0.0010 (10)	0.0147 (10)	0.0066 (10)

### (VI) (2E)-1-(5-Bromothiophen-2-yl)-3-(3-methoxyphenyl)prop-2-en-1-one Geometric parameters (Å, º)

**Table:**

C1—O1	1.230 (2)	C31—C36	1.388 (3)
C1—C12	1.467 (3)	C31—C32	1.402 (3)
C1—C2	1.469 (3)	C32—C33	1.380 (3)
C2—C3	1.331 (3)	C32—H32	0.9500
C2—H2	0.9500	C33—O33	1.368 (2)
C3—C31	1.465 (3)	C33—C34	1.387 (3)
C3—H3	0.9500	C34—C35	1.379 (3)
S11—C15	1.708 (2)	C34—H34	0.9500
S11—C12	1.7206 (19)	C35—C36	1.387 (3)
C12—C13	1.375 (3)	C35—H35	0.9500
C13—C14	1.414 (3)	C36—H36	0.9500
C13—H13	0.9500	O33—C37	1.418 (2)
C14—C15	1.353 (3)	C37—H37A	0.9800
C14—H14	0.9500	C37—H37B	0.9800
C15—Br15	1.8681 (18)	C37—H37C	0.9800
			
O1—C1—C12	119.71 (18)	C32—C31—C3	118.29 (19)
O1—C1—C2	122.72 (19)	C33—C32—C31	120.24 (19)
C12—C1—C2	117.57 (19)	C33—C32—H32	119.9
C3—C2—C1	121.2 (2)	C31—C32—H32	119.9
C3—C2—H2	119.4	O33—C33—C32	124.89 (19)
C1—C2—H2	119.4	O33—C33—C34	114.96 (17)
C2—C3—C31	127.2 (2)	C32—C33—C34	120.15 (18)
C2—C3—H3	116.4	C35—C34—C33	119.68 (18)
C31—C3—H3	116.4	C35—C34—H34	120.2
C15—S11—C12	91.15 (10)	C33—C34—H34	120.2
C13—C12—C1	131.11 (18)	C34—C35—C36	120.8 (2)
C13—C12—S11	111.13 (14)	C34—C35—H35	119.6
C1—C12—S11	117.76 (15)	C36—C35—H35	119.6
C12—C13—C14	112.87 (18)	C35—C36—C31	119.76 (19)
C12—C13—H13	123.6	C35—C36—H36	120.1
C14—C13—H13	123.6	C31—C36—H36	120.1
C15—C14—C13	111.69 (18)	C33—O33—C37	117.69 (16)
C15—C14—H14	124.2	O33—C37—H37A	109.5
C13—C14—H14	124.2	O33—C37—H37B	109.5
C14—C15—S11	113.17 (15)	H37A—C37—H37B	109.5
C14—C15—Br15	127.49 (15)	O33—C37—H37C	109.5
S11—C15—Br15	119.32 (11)	H37A—C37—H37C	109.5
C36—C31—C32	119.33 (18)	H37B—C37—H37C	109.5
C36—C31—C3	122.38 (18)		
			
O1—C1—C2—C3	−4.7 (4)	C12—S11—C15—Br15	178.16 (13)
C12—C1—C2—C3	174.42 (19)	C2—C3—C31—C36	1.0 (3)
C1—C2—C3—C31	179.66 (19)	C2—C3—C31—C32	−178.2 (2)
O1—C1—C12—C13	−172.7 (2)	C36—C31—C32—C33	−1.0 (3)
C2—C1—C12—C13	8.2 (4)	C3—C31—C32—C33	178.14 (18)
O1—C1—C12—S11	6.5 (3)	C31—C32—C33—O33	−179.10 (19)
C2—C1—C12—S11	−172.61 (16)	C31—C32—C33—C34	1.1 (3)
C15—S11—C12—C13	0.11 (17)	O33—C33—C34—C35	179.8 (2)
C15—S11—C12—C1	−179.23 (17)	C32—C33—C34—C35	−0.4 (3)
C1—C12—C13—C14	179.2 (2)	C33—C34—C35—C36	−0.4 (4)
S11—C12—C13—C14	0.0 (2)	C34—C35—C36—C31	0.5 (4)
C12—C13—C14—C15	−0.2 (3)	C32—C31—C36—C35	0.2 (3)
C13—C14—C15—S11	0.3 (3)	C3—C31—C36—C35	−178.9 (2)
C13—C14—C15—Br15	−177.96 (15)	C32—C33—O33—C37	7.9 (3)
C12—S11—C15—C14	−0.22 (18)	C34—C33—O33—C37	−172.3 (2)

### (VI) (2E)-1-(5-Bromothiophen-2-yl)-3-(3-methoxyphenyl)prop-2-en-1-one Hydrogen-bond geometry (Å, º)

**Table:**

*D*—H···*A*	*D*—H	H···*A*	*D*···*A*	*D*—H···*A*
C13—H13···O1^i^	0.95	2.54	3.446 (3)	159

Symmetry code: (i) −*x*+1, *y*−1/2, −*z*+3/2.
